# CD4 Receptor is a Key Determinant of Divergent HIV-1 Sensing by Plasmacytoid Dendritic Cells

**DOI:** 10.1371/journal.ppat.1005553

**Published:** 2016-04-15

**Authors:** Meagan O’Brien, Olivier Manches, Craig Wilen, Ramya Gopal, Rumana Huq, Vernon Wu, Nicole Sunseri, Nina Bhardwaj

**Affiliations:** 1 Division of Infectious Diseases, Department of Medicine, Icahn School of Medicine at Mount Sinai, New York, New York, United States of America; 2 Division of Hematology and Oncology, Hess Center for Science and Medicine, Icahn School of Medicine at Mount Sinai, New York, New York, United States of America; 3 Department of Pathology & Immunology, Washington University School of Medicine, St. Louis, Missouri, United States of America; 4 Microscopy Shared Resource Facility, Icahn School of Medicine at Mount Sinai, New York, New York, United States of America; 5 Department of Pediatrics, the University of Chicago, Chicago, Illinois, United States of America; University of Wisconsin, UNITED STATES

## Abstract

Plasmacytoid dendritic cells (pDC) are innate immune cells that sense viral nucleic acids through endosomal Toll-like receptor (TLR) 7/9 to produce type I interferon (IFN) and to differentiate into potent antigen presenting cells (APC). Engagement of TLR7/9 in early endosomes appears to trigger the IRF7 pathway for IFN production whereas engagement in lysosomes seems to trigger the NF-κB pathway for maturation into APC. We showed previously that HIV-1 (HIV) localizes predominantly to early endosomes, not lysosomes, and mainly stimulate IRF7 rather than NF-κB signaling pathways in pDC. This divergent signaling may contribute to disease progression through production of pro-apoptotic and pro-inflammatory IFN and inadequate maturation of pDCs. We now demonstrate that HIV virions may be re-directed to lysosomes for NF-κB signaling by either pseudotyping HIV with influenza hemagglutinin envelope or modification of CD4 mediated-intracellular trafficking. These data suggest that HIV envelope-CD4 receptor interactions drive pDC activation toward an immature IFN producing phenotype rather than differentiation into a mature dendritic cell phenotype.

## Author Summary

Plasmacytoid dendritic cells (pDC) are innate immune cells that are specialized to produce type I interferon (IFN) and to activate adaptive immune responses. Although IFN is an anti-viral cytokine, it may contribute more to pathogenesis than to protection during chronic viral infections, including chronic HIV infection. pDC sense HIV to produce abundant IFN but minimal NF- κB–dependent production of TNFα and minimal up-regulation of co-stimulatory molecules, suggesting that HIV promotes pDC to become interferon producing cells (IPC) rather than antigen presenting cells (APC). Here, we use florescent HIV virions pseudotyped with influenza hemagglutinin (HA) envelope and a cell system expressing CD4 molecules with modified intracellular trafficking. We found that HIV virions pseudotyped with HA stimulate pDC to mature, similar to influenza-stimulated pDC, and traffic intracellularly similarly to influenza. We also find that CD4-mediated intracellular trafficking guides HIV trafficking and downstream signaling. Our study presents new and important findings which demonstrate that divergent HIV sensing by pDC to produce IFN, rather than to become mature antigen presenting cells, is mediated specifically by CD4-HIV envelope interactions.

## Introduction

Type I interferon (IFN) plays a dichotomous role in chronic viral infections such as Human Immunodeficiency Virus-1 (HIV), contributing to the control of viral replication during the earliest stages of infection, yet fueling disease progression by activating target cells for infection, decreasing antiviral gene expression, enabling infection with increased reservoir size, and accelerating CD4 T-cell loss [1–8]. Plasmacytoid dendritic cells (pDC) are thought to play a significant role in IFN responses during HIV infection, arriving rapidly at sites of mucosal transmission [4] and relocating from blood to lymphoid tissues where they produce pro-apoptotic and pro-inflammatory IFN [9–11].

Cellular mechanisms underlying HIV-stimulated IFN production by pDC are only partially understood. We have previously shown that abundant IFN is produced by pDC upon HIV stimulation through endosomal recognition of genomic RNA by TLR7. This response requires the presence of HIV envelope protein on viral particles, interactions between CD4 and the viral envelope protein, HIV endocytosis and endosomal acidification; however, co-receptor usage, viral fusion and viral replication are not required [12, 13]. Cell-to-cell infection seems to amplify pDC responses to HIV, however precise mechanisms underlying differences between cell-free and cell-to-cell pDC activation are not clearly defined [14]. We and others have shown that pDC are highly resistant to HIV infection, and this block to replication is IFN-independent [15, 16].

In addition to IFN production, pDC can act as antigen-presenting cells (APC) to activate T-cell–mediated adaptive immune responses [17–21]. Acquisition of an APC phenotype requires specific signals that are distinct from the signals that induce large amounts of IFN. We have previously shown that HIV stimulated pDC express low levels of the co-stimulatory molecule CD86 and express Indoleamine 2,3-dioxygenase (IDO), a potent inducer of regulatory T cells, indicating that they do not differentiate into mature APC and fail to stimulate potent T cell responses [22, 23]. However, pDC can differentiate into APC with influenza virus or the synthetic TLR7 agonist R837 and are able to cross-present antigens from HIV-1-infected apoptotic cells to HIV-specific CD8+ T lymphocytes, demonstrating that pDC do not have an intrinsic defect in presentation of HIV antigens, but rather that sensing of HIV does not provide the signals that are required for efficient differentiation of pDC into APC [17].

pDC sense single stranded RNA or unmethylated DNA containing Cytosine–Guanosine dinucleotides (CpG) through Toll-like receptors (TLR) 7 and 9, respectively, located in endosomal compartments. Both TLR7 and TLR9 signal through the adapter protein myeloid differentiation primary response gene 88 (MyD88). Downstream IFN signaling occurs in response to activation of IFN genes through phosphorylation of interferon regulatory factor 7(IRF7), whereas downstream signaling of nuclear factor kappa-light-chain-enhancer of activated B cells (NF-κB) leads to the transcriptional activation of proinflammatory kinases and upregulation of MHC and co-stimulatory molecules necessary for maturation into APC. [12, 24].

The functional response of pDC to pathogens is flexible. As posited by the spatiotemporal model of pDC sensing [25], differential pDC activation is likely related to the subcellular location where the TLR senses the pathogen. Thus, engagement of TLR 7/9 in the early endosomes of pDC preferentially triggers the IRF7 signal cascade, leading to type I IFN responses; whereas engagement of TLR7/9 in lysosomes preferentially triggers the NF-κB signal cascade, leading to the production of proinflammatory cytokines TNFα and IL6, upregulation of co-stimulatory molecules, and an APC phenotype [25, 26]. Differential trafficking and therefore sensing of synthetic TLR9-activating CpGs is attributed to sequence-related secondary and tertiary structural features of the CpGs. CpGs which contain phosphodiester backbones and palindromic motifs (CpGA) form multimeric complexes and traffic to early endosomes for IRF7 signaling whereas CpGs which contain phosphorothioate backbones and lack palindromic motifs (CpGB) traffic as monomers to lysosomes for NF-κB signaling. Intermediate CpGs (CpGC) combine structural elements of both CpGA and CpGB, traffic to both compartments, and stimulate both IRF7 and NF-κB signaling [27–29].

While the spatiotemporal model of pDC sensing has been most clearly evaluated using synthetic TLR9 agonists (CpG), we have shown that the model also applies to HIV and TLR7, whereby HIV traffics to early endosomes in pDC, activating IRF7 signaling rather than NF-κB signaling [22, 23]. The upstream events that determine activation of each of these pathways, and in particular, HIV virion trafficking in pDC, are currently unknown, however, prior studies suggest that HIV envelope may play a major role [13, 30, 31]. Here we demonstrate that HIV trafficking and pDC phenotype is predominantly determined by envelope-CD4 interaction, such that manipulation of HIV envelope or CD4 intracellular trafficking enables modulation of divergent sensing of HIV.

## Results

### Viral envelope directs intracellular trafficking of HIV virions in pDC and resultant phenotype

**Fig 1:**
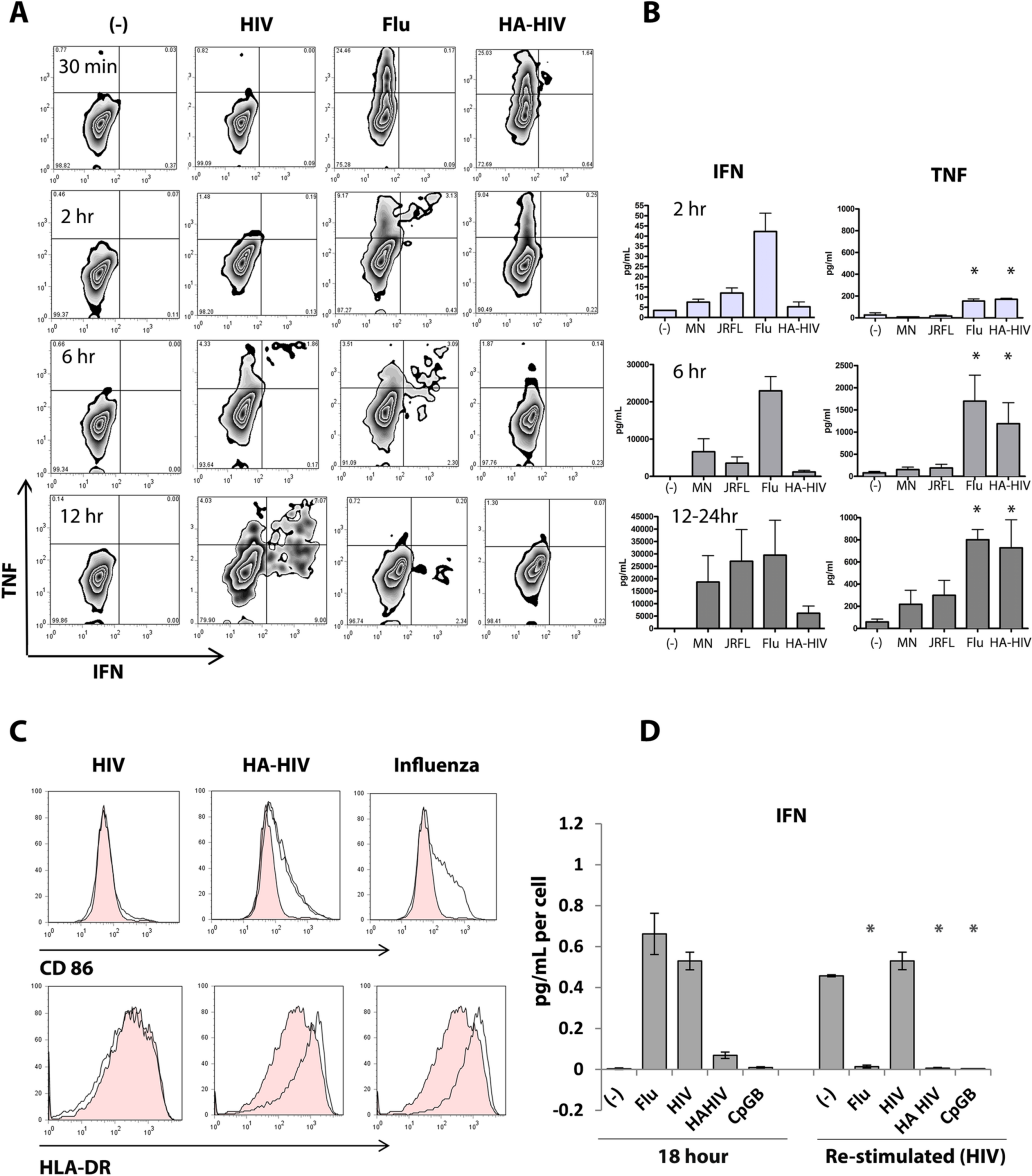
HIV pseudotyped with influenza hemagglutinin envelope activates signaling pathways in pDC similarly to influenza. Human purified pDC incubated with virions. Data are from 1 experiment, representative of 3 independent experiments. (A) pDC were incubated with media (-), MN HIV, influenza (Flu), or HA-HIV for 24 hours and TNF and IFN production were detected by ICS at time points as shown by FACS plot. (B) pDC were incubated with media(-), MN HIV, JRFL HIV, influenza (Flu), or HA-HIV at 50,000 cells/100uL and supernatants were tested for soluble IFN and TNF at 2, 6, and 12–24 hours, where Flu and HA-HIV are compared to JRFL HIV, unpaired Student’s t test *p<0.05. (C) FACS plot demonstrating maturation as assessed by CD86 and HLA-DR expression, unstimulated cells (filled histogram), stimulated cells (open histogram). (D) pDC were incubated overnight with media (-), influenza (Flu), HIV, HA-HIV, or CpGB, culture supernatants were collected, cells were washed, and cells were incubated for a second overnight stimulation with HIV and culture supernatants were collected. IFN was measured in the culture supernatants by ELISA and corrected for cell number. Bar graphs represent 3 experiments with mean ± SEM. When initially stimulated with HIV, pDC may be re-stimulated to produce IFN, upon subsequent HIV stimulation (4.24 pg/ml per cell ± 1.13 pg/ml per cell). In comparison, when pDC are initially stimulated with Flu, HA-HIV, or CpGB, respectively, pDC cannot be re-stimulated by HIV to produce IFN(0.10 pg/ml per cell ± 0.03 pg/ml per cell, 0.05 pg/ml per cell ± 0.01 pg/ml per cell, 0.04 pg/ml per cell ± 0.01 pg/ml per cell), unpaired Student’s t test *p<0.05.

We hypothesized that HIV envelope protein interactions with cell surface CD4 determine the intracellular trafficking of HIV and the resultant signaling in pDC, based on the spatiotemporal model of TLR signaling [25]. To test this, we replaced HIV envelope protein with envelope protein from a virus that activates pDC to differentiate into mature pDC, namely influenza virus. Influenza virus hemagglutinin envelope glycoprotein (HA) binds to sialic acids on the cell surface to trigger clathrin-dependent endocytosis [32]. We pseudotyped HIV virions with influenza hemagglutinin glycoprotein envelope (HA-HIV) and first compared the functional response of purified pDC to HIV, influenza, and HA-HIV, in terms of magnitude and kinetics of TNFα (TNF) and IFN production. TNF is produced downstream of NF-κB signaling and IFN is produced downstream of IRF7 signaling. TNF and IFN were measured by intracellular cytokine staining (ICS) at 30 minutes, 2 hours, 6 hours, and 12 hours and in the culture medium by cytokine bead array (CBA) and ELISA, respectively, at 2 hours, 6 hours, and 12–24 hours. As previously demonstrated [22], the response of pDC to HIV was characterized by delayed IFN and TNF responses with IFN predominating at later time points. In comparison, both HA-HIV and influenza stimulated pDC to rapidly produce TNF within 30 minutes- 2 hours, an effect which plateaued by 12–24 hours. Influenza also stimulated early IFN secretion, within 2 hours. HA-HIV induced IFN secretion, albeit at lower levels than Flu itself (Fig 1A and 1B), possibly due to faster trafficking kinetics and early global cytokine shutdown (as evidenced by earlier IFN and TNF shutdown compared to Flu in ICS Fig 1A). Strikingly, TNF was always produced antecedent to IFN, as evidenced by ICS staining, and as has been observed in murine pDC [33] (Fig 1A). After overnight incubation of pDC, HIV stimulated minimal upregulation of CD86 and HLA-DR while HA-HIV and influenza stimulated strong upregulation of CD86 and HLA-DR expression, providing further evidence that HA-HIV and influenza activate NF-κB signaling/maturation pathways in pDC while HIV does not (Fig 1C). Similar maturation and IFN effects were seen whether pDC were stimulated with X4 lab strain, MN or HIV backbone pNL43-ΔEnv-vpr+-luc+ pseudotyped with X5 HIV envelopes (JRFL, REJO, JOTO) as compared to HIV backbone pNL43-ΔEnv-vpr+-luc+ pseudotyped with hemagglutinin envelopes H1 and H5 (S1A Fig).

A functional measure of pDC maturation is to test whether cells become refractory to re-stimulation by TLR agonists, known as TLR tolerance. We had previously shown that HIV-activated pDC maintain an immature phenotype and are not refractory to re-stimulation to produce IFN. This effect was not due to activation of pDC that had failed to become activated during the previous overnight incubation [22]. We therefore compared the effects of HIV, influenza, HA-HIV, and CpGB, a potent pDC TLR9 maturation stimulus, to inhibit re-stimulation, as a marker of complete pDC maturation. As compared to HIV, HA-HIV inhibited re-stimulation of pDC similarly to influenza and CpGB, thus signifying that HIV pseudotyped with HA matured pDC fully (Fig 1D). Overall, swapping HIV envelope with influenza envelope induced a mature pDC phenotype similar to that induced by influenza activation. Notably, MN HIV, a CXCR4 lab strain of HIV was used in these experiments. However, HIV (pNL43-ΔEnv-vpr+-luc+) pseudotyped with envelopes JRFL, REJO, or JOTO (all R5-tropic) all stimulate pDC to produce IFN, and not to mature, as expected since co-receptor usage is not essential for pDC sensing of HIV (S1A and S1B Fig) [12].

**Fig 2:**
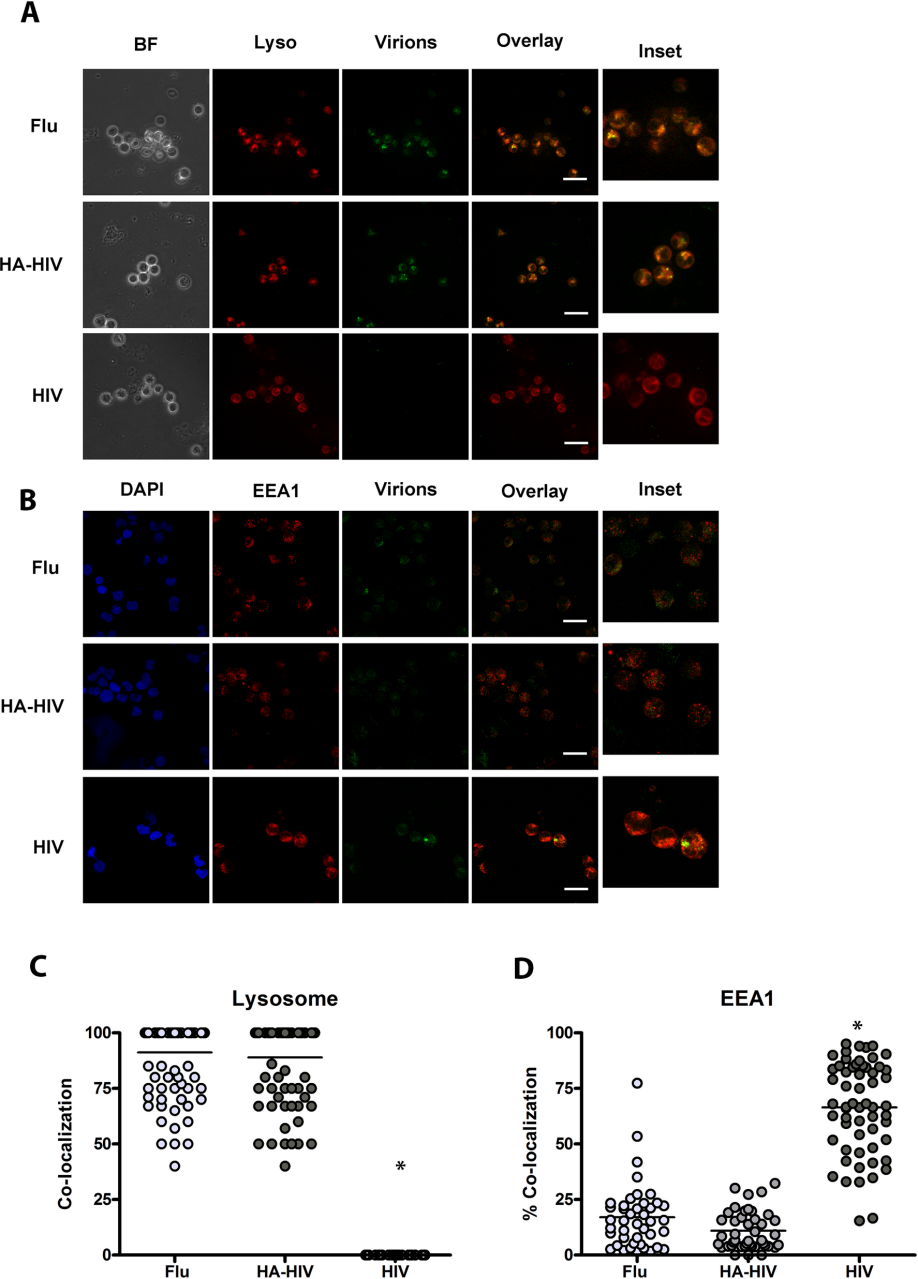
Viral-envelope directs intracellular trafficking of HIV virions in pDC. Human purified pDC 30 minutes after incubation with GFP-virions. (A) Images from live microscopy showing representative brightfield images(BF) and staining for lysotracker (Lyso) of cells incubated with GFP-influenza (Flu), GFP-HA-HIV (HA-HIV), or GFP- JRFL (R5) HIV (HIV) for a single confocal z stack, with overlay of paired images, scale bar = 20μm and inset (3X). (B) Graphs depict % colocalization shown for lysotracker/lysosome (Manders’ coefficient) for 100 cells with mean ±SD comparing Flu 91.24% ±15.15% to HA-HIV 88.94% ± 17.59% to HIV 0.00% ± 0.00%, unpaired Student’s t test comparing Flu to HIV and HA-HIV to HIV, *p<0.001. Data representative of 3 experiments, Magnification X 63. (C-D) Human purified pDC 18 hours after incubation with GFP-virions. Confocal images showing representative staining of pDCs for DAPI and EEA1 when incubated with GFP-influenza (Flu), GFP-HA-HIV (HA-HIV), or GFP-HIV (HIV) for a single confocal z stack with overlay of paired images, scale bar = 20μm and inset (3X). (D) Bar graphs depict % colocalization (Manders coefficient) of virions with EEA1 with mean ± SD for 50 cells comparing Flu 17.03% ±14.82% to HA-HIV 10.99% ±8.66% to HIV 66.42% ±20.69%, unpaired Student’s t test comparing Flu to HIV and HA-HIV to HIV, *p<0.01. Data representative of 3 experiments. Magnification X100.

Because HA-HIV and influenza similarly stimulated pDC to mature, we sought to investigate whether HA-HIV traffics similarly to influenza in pDC. HIV virions (pNL43-ΔEnv-vpr+-luc+) pseudotyped with HA and packaging green florescent protein (GFP) and HIV virions (pNL43-ΔEnv-vpr+-luc+) pseudotyped with JRFL (R5 envelope) and packaging GFP were generated, using eGFP-Vpr plasmids, as previously described [22]. Influenza packaging GFP were also generated, as previously described [34, 35]. We found that HA-HIV, similarly to influenza and unlike HIV itself, rapidly trafficked to lysosomes by 30 minutes as evidenced by co-localization with Lysotracker, a dye that traffics to these organelles (Fig 2A and 2C). Both influenza and HA-HIV extensively co-localized with Lysotracker at 30 minutes and 2–4 hours, whereas HIV was barely visible inside the cell at these early time points. After overnight incubation HIV was well visualized inside the cell, but did not co-localize well with lysotracker. Influenza and HA-HIV still seemed to co-localize with lysotracker even though the florescent signal was faded, likely due to lysosomal degradation of the virions (S2A–S2D Fig). We confirmed that HIV traffics to early endosomal (EEA1) compartments by 18 hours as previously shown [22], whereas influenza and HA-HIV traffic significantly less to these compartments and the green signal is faded at 18 hours (Fig 2B and 2D). Thus, the nature of the viral envelope seems crucial to determining trafficking of virions in pDC, and for the downstream signaling pathways activated in different intracellular compartments.

### CD4 trafficking guides HIV intracellular tropism

Our results indicate that viral envelope protein dictates early intracellular trafficking of virions in pDC, suggesting that trafficking is directed by interaction of HIV envelope protein with its cognate receptor. In pDC, sensing of HIV involves CD4-mediated endocytosis [12]. CD4 is a type I integral membrane glycoprotein that can be internalized through clathrin-mediated endocytosis. The intracellular tail of CD4 displays motifs important for its internalization: a dileucine motif that allows interaction with the clathrin adaptor 2 (AP-2) [36] and an adjacent serine, whose phosphorylation augments the affinity of the dileucine motif for AP-2 [36], thereby regulating CD4 endocytosis. In cells of macrophage-monocyte lineage, CD4 is constitutively endocytosed at low levels through clathrin-coated pits to early and recycling endosomes [37], as CD4 is serine-phosphorylated to some extent even in unstimulated cells [36]. We first examined whether CD4 itself and CD4-associated targeting motifs are responsible for the predominant localization of HIV in early endosomes. We used HEK 293 T (HEK) cells and HEK reporter cells as a model because HEK cells do not express CD4 under native conditions and therefore manipulation of CD4 trafficking and viral-CD4 interactions can be studied more clearly. Moreover, due to technical limitations, it was not possible to transfect or transduce primary pDCs or the Gen2.2 pDC cell line to undertake these studies.

**Fig 3:**
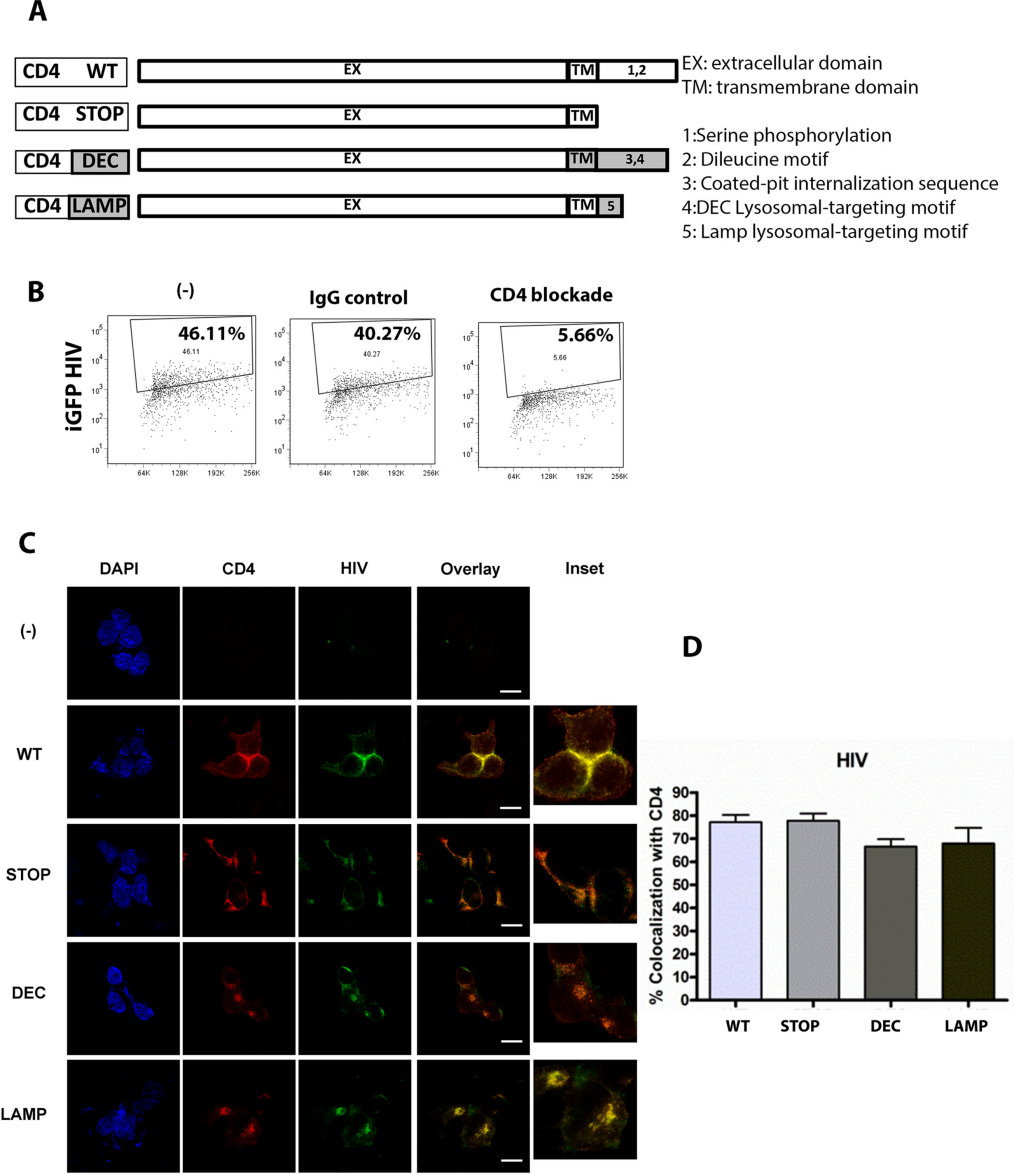
HIV colocalizes with CD4 in CD4-transduced HEK cells. (A) CD4 mutants were constructed as depicted. (B) FACS plot of CD4-transduced HEK cells incubated with GFP HIV (-) for 2–4 hours in the presence or absence of CD4 blockade or IgG control antibody; gaiting was set for uninfected cells (C) Confocal images showing representative staining of CD4-transduced HEK cells incubated with GFP-HIV for 2 to 4 hours for CD4 and GFP HIV for a single confocal mid-plane z stack with overlay of paired images, scale bar = 20μm and inset (3X), where (-) is HEK not transduced with CD4, WT is CD4-WT, STOP is CD4-STOP, DEC is CD4-DEC, LAMP is CD4-LAMP (D) Bar graphs depict % colocalization (Manders coefficient) of GFP HIV (HIV) with CD4 with mean ± SD (WT 77.12± 12.34, STOP 77.75± 11.83, DEC 66.58± 20.62, LAMP 67.92± 26.20) for 50 cells, unpaired Student’s t-test comparing WT to each group, with no significant difference between groups. Data representative of 3 experiments. Magnification X100.

We engineered hybrid CD4 molecules, mutating its intracytoplasmic tail or swapping it with the intracellular domain of CD205 (DEC205) and Lamp-1 (Fig 3A). CD205 and Lamp-1 contain distinct lysosome-targeting motifs in their intracytoplasmic tail that induce constitutive targeting to late endosomes/lysosomes [38–40]. DEC-205 expresses the coated-pit internalization sequence (FSSVRY) and lysosome-targeting motif (EDE), whereas Lamp-1 expresses the lysosomal targeting motif (YQTI). Several mutants were tested, as visualized in Fig 3A: (1). CD4-WT (wild type CD4), (2). CD4-STOP (lacking the cytoplasmic domain), (3). CD4-DEC (replacing the CD4 cytoplasmic domain with the DEC-205 cytoplasmic domain to shuttle CD4 to the lysosomes), and (4). CD4-LAMP (replacing the CD4 cytoplasmic domain with the Lamp-1 cytoplasmic domain to shuttle CD4 to the lysosomes). The CD4 mutant sequences were introduced into lentiviral vectors for stable transduction of HEK cells for microscopy, and HEK-Blue hTLR7-expressing cells to measure NF-κB activation by HIV. HEK-Blue hTLR7 co-express human TLR7 and an NF-κB inducible secreted embryonic alkaline phosphatase (SEAP) reporter gene. CD4 expression was maintained in the presence of puromycin, and CD4 expression across cell lines was uniform at 65–75%. For these experiments we used JRFL HIV packaging GFP- HIV Gag-iGFP (GFP HIV) to track HIV intracellular trafficking. Following incubation with HIV for 2 to 4 hours, CD4-expressing cells bound and endocytosed HIV efficiently, as shown in Fig 3C. This time point was chosen because HIV was not visualized well before 2 hours, and the fluorescent signal was faded after overnight incubation. The main path of viral entry in CD4-expressing HEK cells is CD4-mediated endocytosis as CD4 blockade completely abrogated HIV uptake (Fig 3B). HIV co-localized extensively with CD4, whatever CD4 construct the cells expressed, in the range of 65% to 80% co-localization per cell, as measured by single cell Mander’s coefficient (Fig 3D). HEK cells which are not expressing CD4 do not take up HIV as represented by the (-) condition in representative images (Fig 3C). Although the intracellular distribution pattern appeared different between the different CD4 constructs, with CD4-WT and CD4-STOP appearing more cell-surface associated and CD4-LAMP and CD4-DEC appearing more internal compartment associated, the overall fluorescence intensities of cell-associated HIV were comparable.

**Fig 4:**
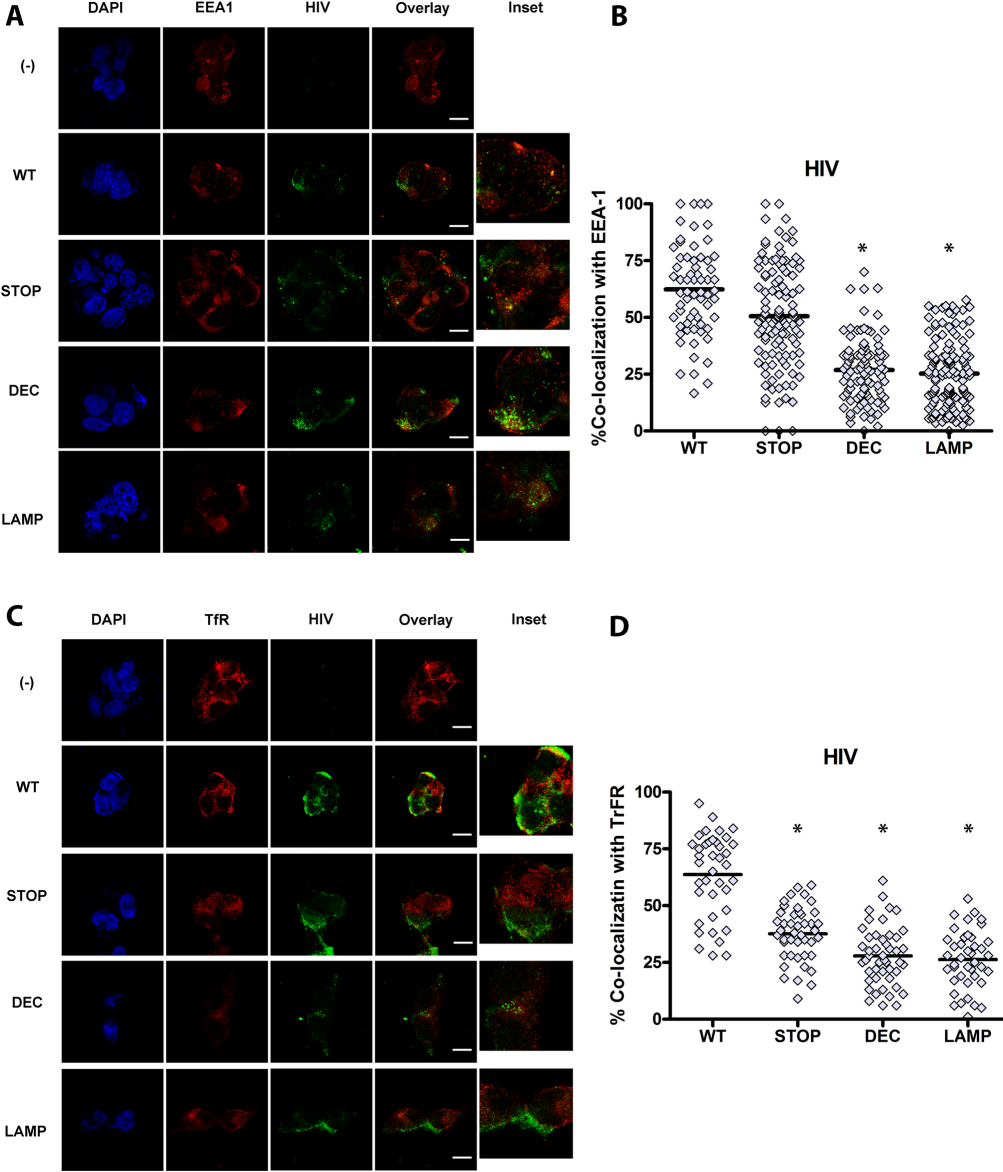
CD4 (WT) intracellular trafficking guides HIV intracellular localization to early and recycling compartments. CD4-transduced HEK cells incubated with GFP HIV for 2–4 hours. Confocal images showing representative staining of CD4-transduced HEK cells, where(-) is HEK not transduced with CD4, WT is CD4-WT, STOP is CD4-STOP, DEC is CD4-DEC, LAMP is CD4-LAMP incubated with GFP HIV for (A) EEA1 and (C) TfR with GFP HIV for a single confocal z stack with overlay of paired images, scale bar = 20μm and inset (3X). Graphs depict % colocalization (Manders coefficient) for 50–100 cells of GFP HIV (HIV) with (B) EEA-1 mean ± SD in CD4-WT cells (WT) 62.39% ± 20.31%, in CD4-STOP cells (STOP) 50.52% ± 22.15%, in CD4-DEC cells (DEC) 26.84% ± 14.35%, and in CD4-LAMP cells (LAMP) 25.29% ± 14.92%, * unpaired Student’s t test p<0.001, comparing WT to STOP, WT to DEC, and WT to LAMP. (D) % colocalization of HIV with TfR mean ± SD in WT 63.67% ± 18.38%, in STOP 37.61% ±11.92%, in DEC 27.87% ± 12.95%, in LAMP 26.32% ± 12.77%, unpaired Student’s t test *p<0.001 Data representative of 3 experiments. Magnification X100.

**Fig 5:**
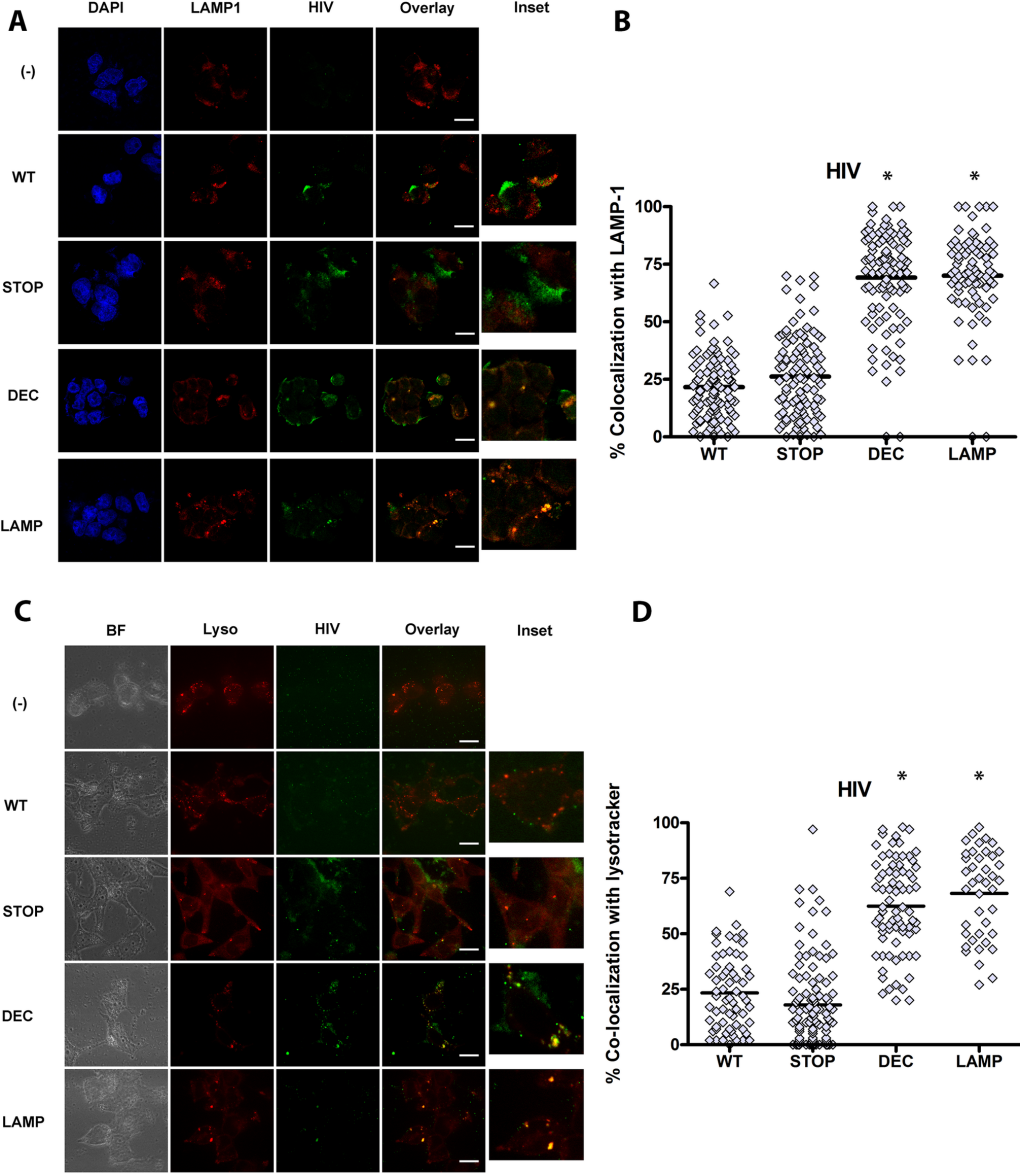
CD4 (LAMP and STOP) intracellular trafficking guides HIV intracellular localization to lysosomal compartments. CD4-transduced HEK cells incubated with GFP HIV for 2–4 hours. Confocal images showing representative staining of CD4-transduced HEK cells, where(-) is HEK not transduced with CD4, WT is CD4-WT, STOP is CD4-STOP, DEC is CD4-DEC, LAMP is CD4-LAMP incubated (A) LAMP-1 and (C) lysotracker with GFP HIV for a single confocal z stack with overlay of paired images, scale bar = 20μm and inset (3X). Graphs depict % colocalization (Manders coefficient) for 50–100 cells of GFP HIV (HIV) with (B) LAMP-1 mean ± SD WT 21.59% ± 13.62% as compared to STOP 26.21% ±17.71% as compared to DEC 69.25% ± 20.70% as compared to LAMP 70.01% ± 18.93%, unpaired Student’s t test *p<0.01, comparing WT to STOP, WT to DEC, and WT to LAMP. (D) % colocalization of HIV with lysotracker mean ± SD in WT 23.35% ± 16.64%, in STOP 17.93% ±19.36%, in DEC 62.45% ± 20.84%, in LAMP 68.21% ± 19.67%, unpaired Student’s t test *p<0.01 comparing WT to STOP, WT to DEC, and WT to LAMP.

To better characterize the intracellular localization of GFP- HIV in HEK cells expressing CD4 hybrids, cells were exposed to GFP-HIV for 2–4 hours, then fixed and stained for the early endosomal marker EEA1, the recycling endosomal marker transferrin receptor (TfR), or the lysosomal marker LAMP1. As an additional lysosomal marker, after 2–4 hours of GFP-HIV incubation, lysotracker was added to culture media, and live imaging was performed. Internalized HIV trafficked predominantly to early EEA1+ and TfR+ endosomes in CD4-WT, similar to trafficking of HIV in pDC (Fig 4). However, HIV trafficked mainly to LAMP-1+ and lysotracker+ lysosomes in CD4-DEC and CD4-LAMP (Fig 5). Surprisingly HIV trafficked to EEA1+ compartments in CD4-STOP instead of remaining on the cell surface, but did not traffic predominantly to TfR (Fig 4A–4D). As endosomes containing EEA1 ultimately direct trafficking to the degradative machinery of the cell whereas endosomes containing TfR do not [41], it is likely that some of the mutant CD4-STOP proteins are targeted for degradation to some extent. Overall, these data are consistent with the expected trafficking patterns of each CD4 construct, and indicate that replacing the intracellular tail of CD4 with those of CD205 or LAMP-1 targets CD4 and HIV to the late endosomes/ lysosomes, demonstrating that targeting motifs in CD4 drive the intracellular localization of HIV via CD4-mediated endocytosis.

**Fig 6:**
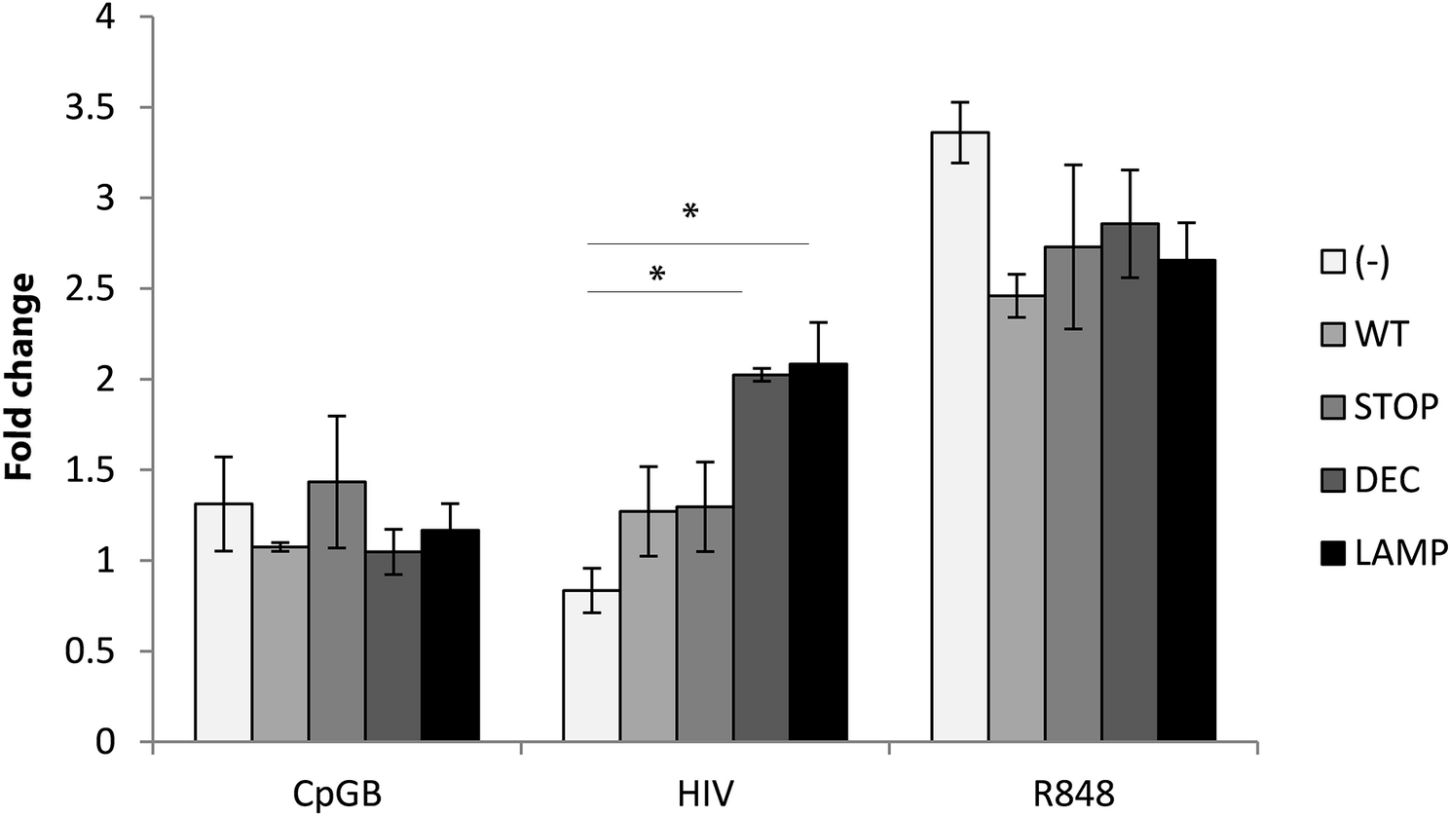
CD4 intracellular trafficking guides activation of signaling pathways by HIV. Bar graphs represent 3 experiments with mean ± SD of CD4-transduced HEK-blue hTLR7 cells incubated with CpGB, HIV, or R848 overnight with NF-kB activation expressed as fold change of O.D. over O.D. of unstimulated cells. Effect of HIV on HEK cells not expressing CD4 (-) 0.83±0.12 as compared to HEK cells expressing WT CD4 1.27±0.24 as compared to HEK cells expressing STOP CD4 1.29.± 0.25 as compared to HEK cells expressing DEC CD4 2.02±0.04 as compared to HEK cells expressing LAMP CD4 2.08±0.23, unpaired Student’s t test *p<0.05).

We next examined the functional consequences of CD4 altered trafficking. The lentiviral constructs (Fig 3A) were transduced into HEK-Blue cells to measure NF-κB activation. Notably this cell type is not optimized to produce IFN, therefore only the NF-κB signaling arm was tested in this system. R848, a TLR7/8 agonist, was used as a positive control and strong NF-κB activator that rapidly accumulates in late endosomes [42]. The TLR9 agonist CpGB was used as a negative control in these TLR7-expressing cells and did not induce NF- κB activation in any condition (Fig 6). Whereas HIV did not induce NF- κB activation in CD4-WT and CD4-STOP expressing cells, it induced NF-κB activation in CD4-DEC and CD4-LAMP expressing cells. These results support the spatiotemporal model of cell signaling, whereby partition into early or late endosomes regulates IFN vs NF-κB signaling [25]. HIV traffics to early endosomes in pDC and in CD4-WT expressing cells, and induces weak NF-κB signaling, whereas retargeting CD4 to lysosomes allows for NF-κB activation by HIV.

**Fig 7:**
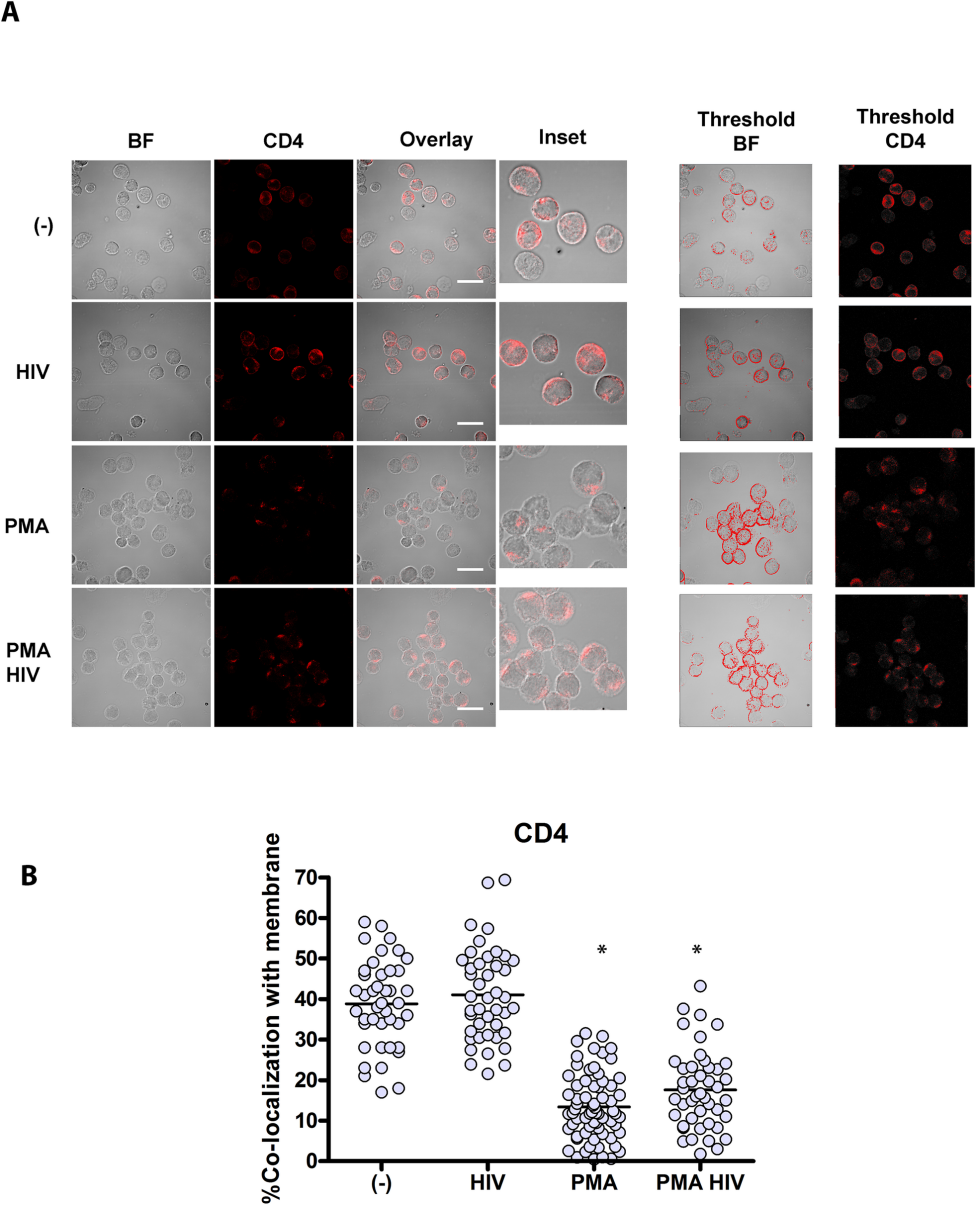
In contrast to PMA stimulation, HIV activation of human pDCs does not cause marked CD4 internalization at early timepoints. (A) Human pDCs incubated with HIV, PMA, or HIV and PMA. Confocal images showing representative cell staining for a single confocal z stack with overlay of paired images, scale bar = 20μm and inset (3X) of CD4 overlaying brightfield images and image analysis by thresholding of membrane region to differentiate membrane localized CD4. Magnification X63 (B) Graphs depict % colocalization (Manders coefficient) of CD4 with membrane by mean ± SD, comparing unstimulated cells (-) 38.83%±10.72% to HIV stimulated 41.03%±11.43% to PMA stimulated 13.37%±8.05%, to HIV+PMA stimulated 17.58%±9.76% unpaired Student’s t test *p<0.01 comparing (-) to PMA and (-) to PMA HIV.

We also examined more precisely why native CD4 delivers HIV into early endosomes. Endocytosis and intracellular CD4 trafficking is dependent on a dileucine motif in its intracellular domain, and is regulated by phosphorylation of an adjacent Serine (Ser408). Phosphorylation of Ser408, as occurs with phorbol ester (PMA), dramatically enhances CD4 delivery from the cell surface to the lysosomes [43]. In contrast to PMA stimulation, HIV activation of human pDCs does not cause marked CD4 internalization at early timepoints. Whether pDC are unstimulated or stimulated with HIV, CD4 internalization is grossly unchanged, whereas PMA stimulation, in the absence or presence of HIV, causes internalization at early timepoints (Fig 7).

**Fig 8:**
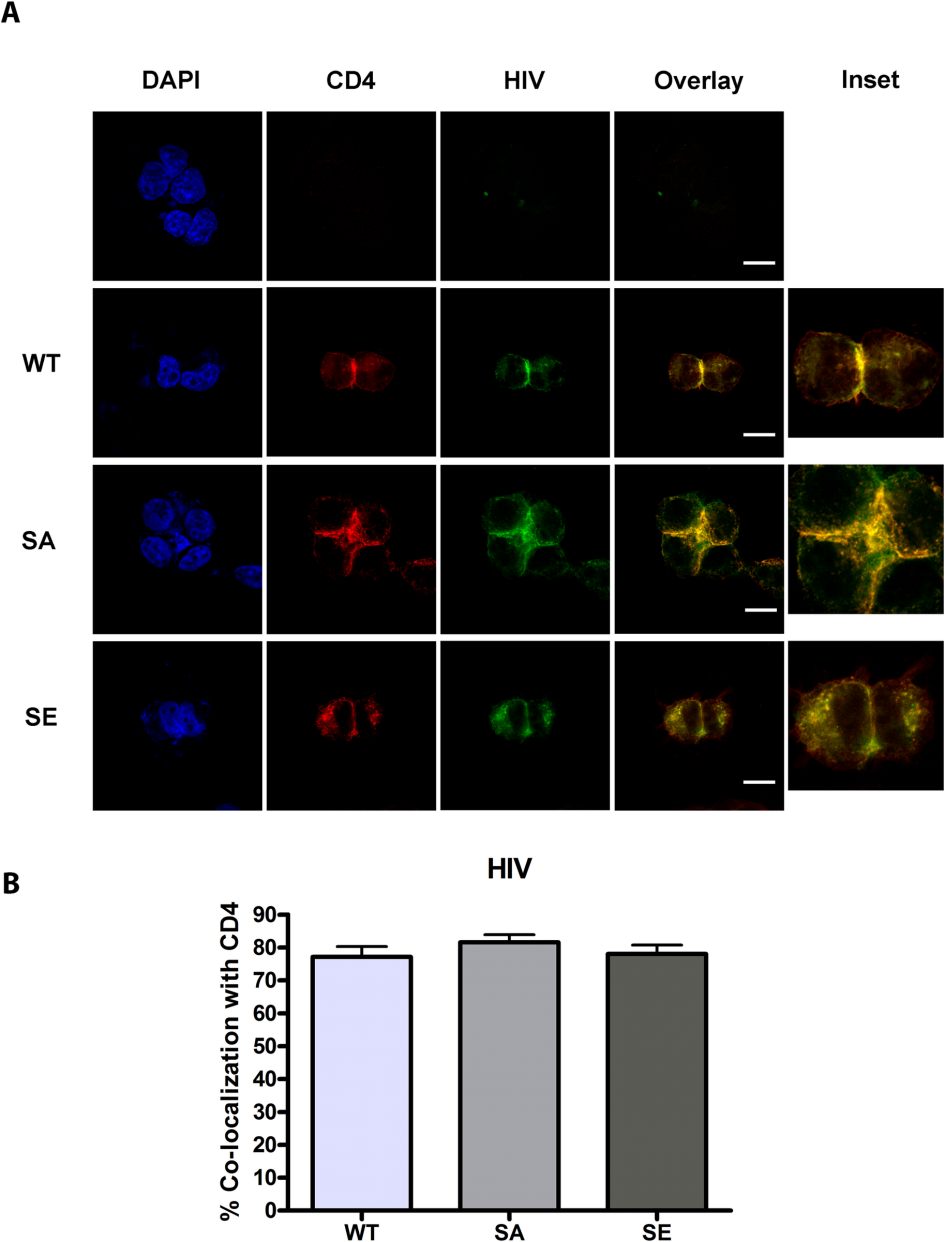
**HIV colocalizes with CD4 in CD4-transduced HEK cells expressing CD4-SA and CD4-SE** (A) Confocal images showing representative staining of CD4-transduced HEK cells incubated with GFP HIV for 2 to 4 hours for CD4 and GFP HIV for a single confocal mid-plane z stack with overlay of paired images, scale bar = 20μm and inset (3X). (B) Bar graphs depict % colocalization (Manders coefficient) of GFP HIV (HIV) with CD4 with mean ± SD (WT 77.12%± 12.34%, SA 81.56%± 11.83%, SE 78.02%± 8.52%) for 50 cells, unpaired Student’s t-test comparing WT to each group, with no significant difference between groups. Data representative of 3 experiments. Magnification X100.

**Fig 9:**
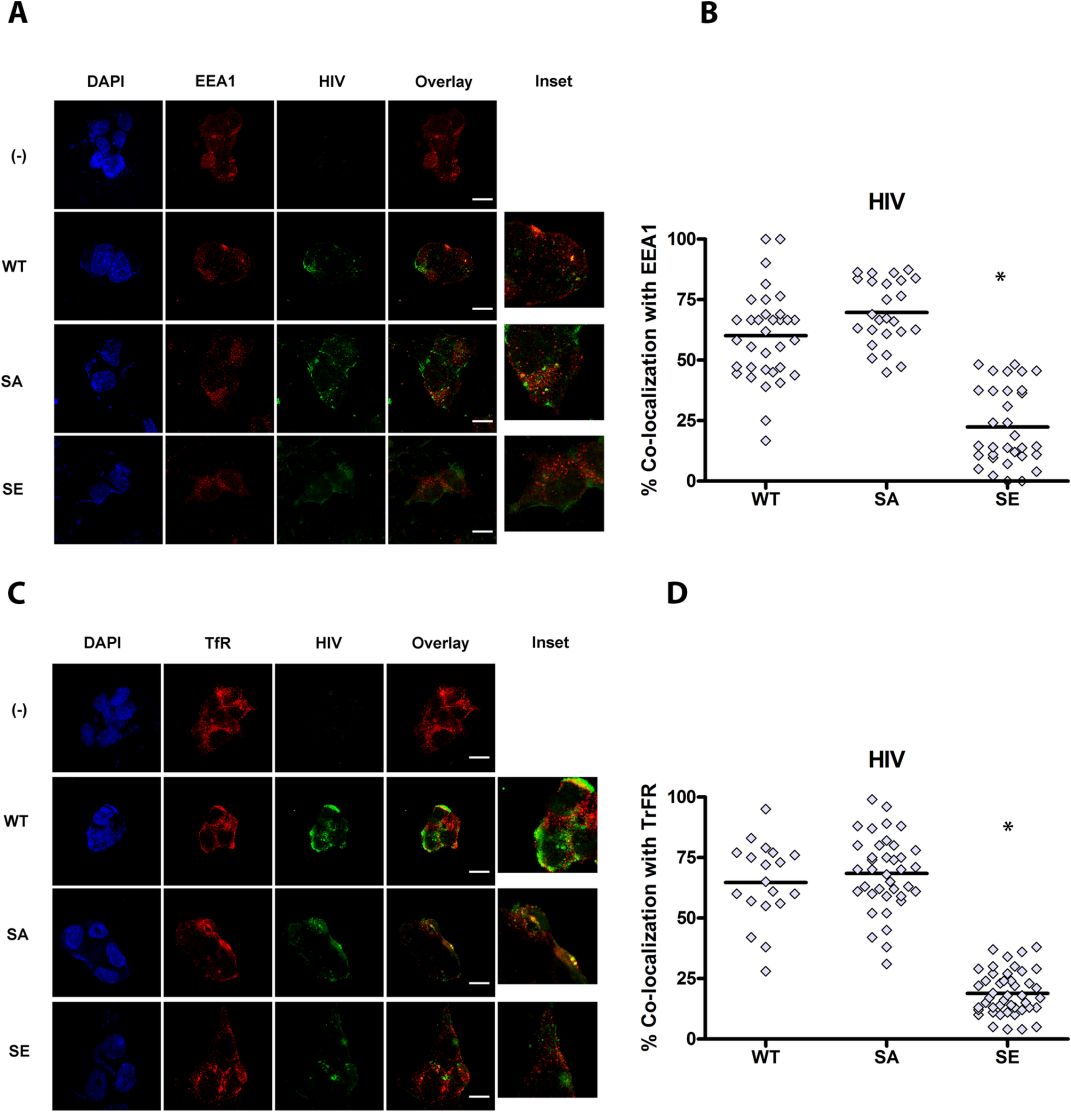
Abrogation of CD4 phosphorylation (SA) targets CD4 and HIV to early/ recycling endosomes. CD4-transduced HEK cells incubated with GFP HIV for 2–4 hours. Confocal images showing representative staining of CD4-transduced HEK cells incubated with GFP HIV for (A) EEA1 and (C) TfR with GFP HIV for a single confocal z stack. Graphs depict % colocalization (Manders coefficient) for 50–100 cells of GFP HIV (HIV) with (B) EEA-1 mean ± SD WT 60.1% ± 18.20% as compared to SA 69.73% ± 13.47% as compared to SE 22.37% ± 16.15%, * unpaired Student’s t test p<0.001, (D) TfR mean ± SD WT 64.68% ± 16.63% as compared to SA 68.45% ±15.48% as compared to SE 18.89% ± 9.00%, unpaired Student’s t test *p<0.01 Data representative of 3 experiments. Magnification X100.

**Fig 10:**
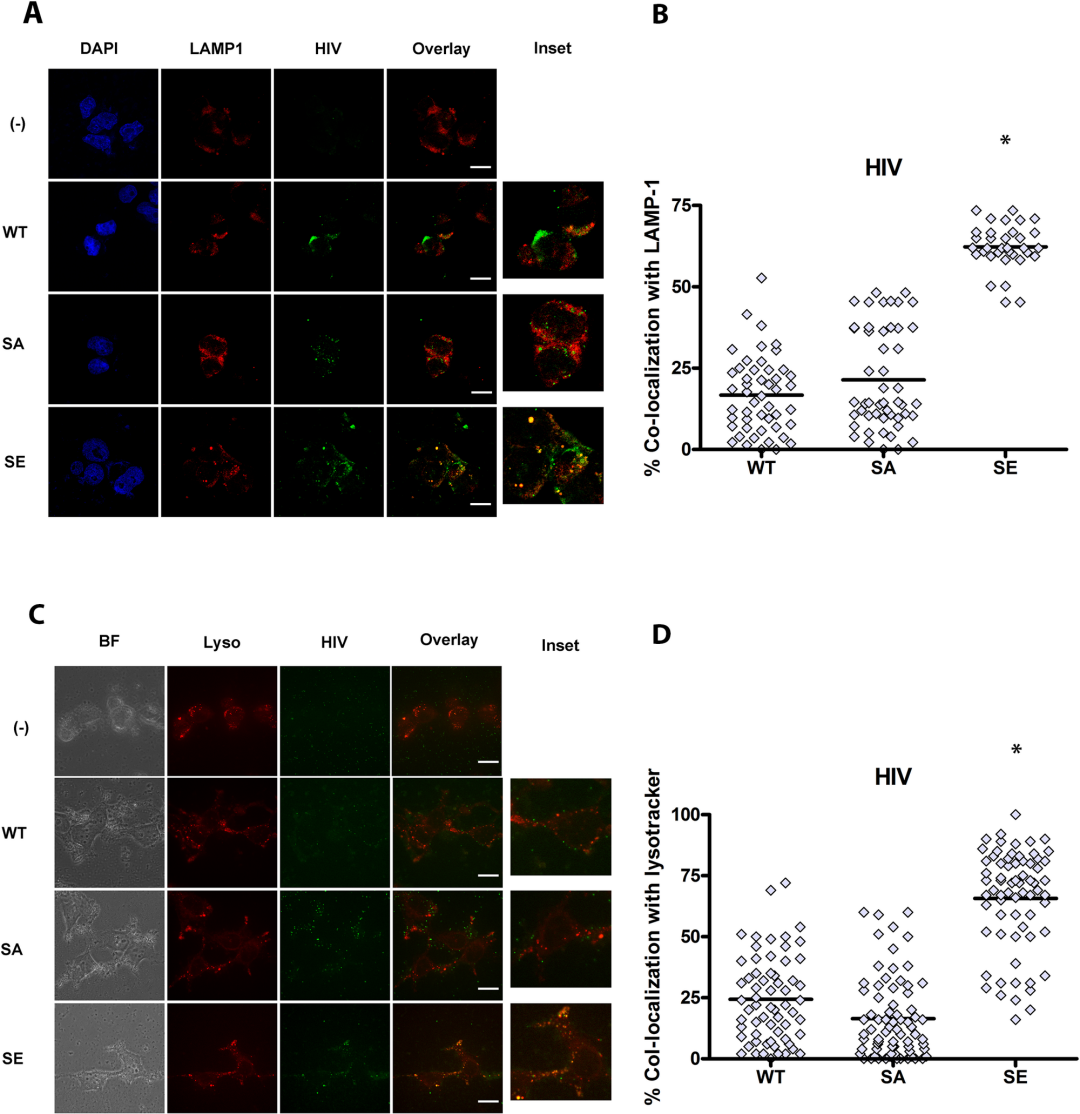
Phospho-mimic of CD4 phosphorylation (SE) targets CD4 and HIV to late endosomes/lysosomes. CD4-transduced HEK cells incubated with GFP HIV for 2–4 hours. Confocal images showing representative staining of CD4-transduced HEK cells incubated with GFP HIV for (A) LAMP-1 or (C) lysotracker with GFP HIV for a single confocal z stack. Graphs depict % colocalization (Manders coefficient) for 50–100 cells of GFP HIV (HIV) with (B) LAMP-1 mean ± SD WT 16.71% ± 11.76% as compared to SA 21.44% ±15.56% as compared to SE 62.27% ± 6.93%, unpaired Student’s t test *p<0.01, (D) Lysotracker mean ± SD WT 24.38% ± 17.60% as compared to SA 16.44% ±16.49% as compared to SE 65.69% ± 20.54%, unpaired Student’s t test *p<0.01. Data representative of 3 experiments. Magnification X100.

**Fig 11:**
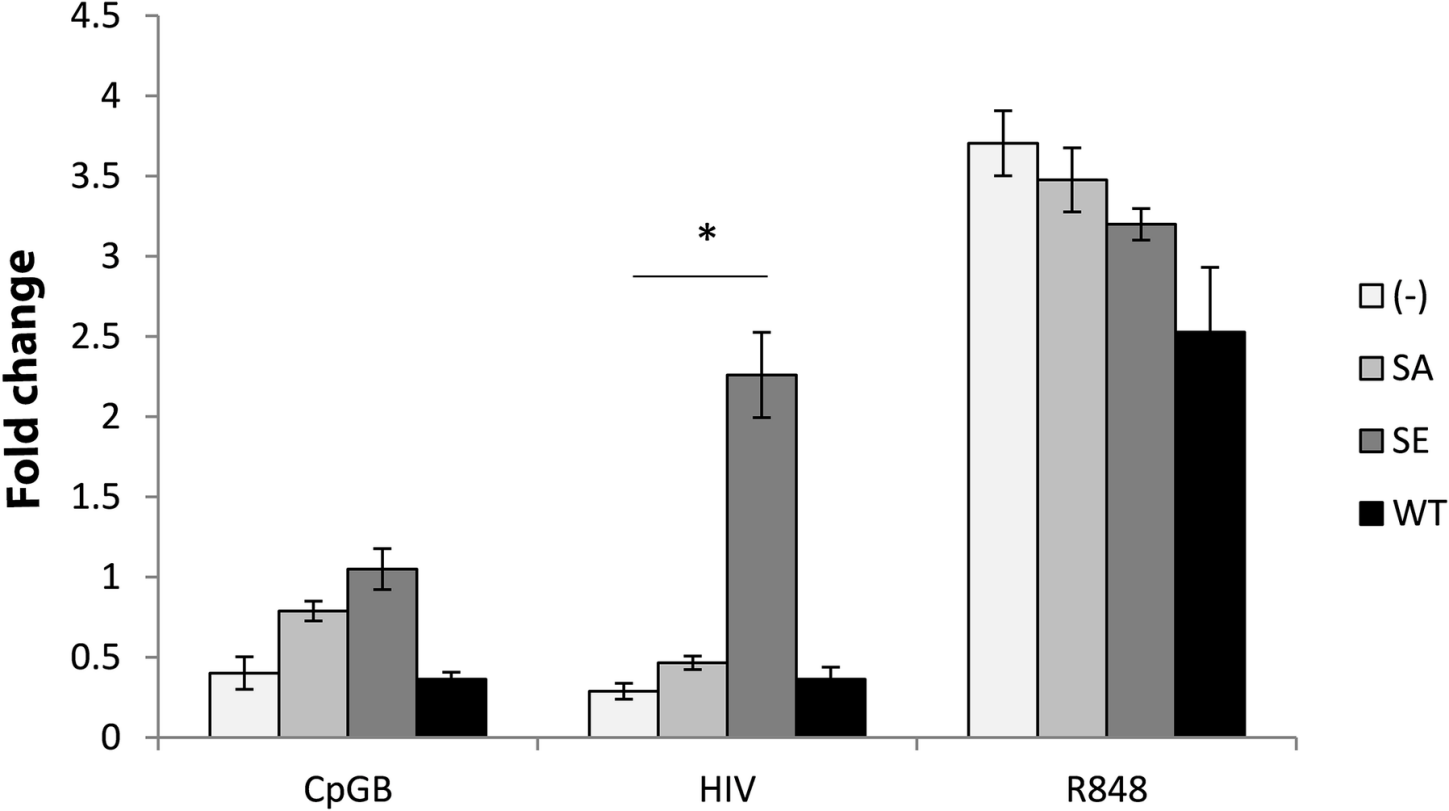
CD4 phosphorylation-guided intracellular trafficking of HIV determines activation of signaling pathways. Bar graphs represent 3 experiments with mean ± SD of CD4-transduced HEK-blue hTLR7 cells incubated with CpGB, HIV, or R848 overnight with NF-kB activation expressed as fold change (O.D.) over unstimulated cells. NF-kB activation is compared between HEK cells not expressing CD4 (-) 0.58±0.05 as compared to HEK cells expressing SA CD4 0.93±0.04 as compared to HEK cells expressing SE CD4 2.26±0.26 as compared to HEK cells expressing WT CD4 0.73±0.0, unpaired Student’s t test *p<0.05.

To investigate whether CD4 phosphorylation on Ser408 targets CD4 and HIV to late endosomes/ lysosomes, we generated two mutations of the serine residue: one to alanine to abrogate phosphorylation (CD4-SA), and one to the phospho-mimic Glutamic Acid (CD4-SE), and produced HEK cells stably expressing these CD4 mutants. Intracellular localization of HIV and CD4 was monitored by microscopy. As shown in Fig 8, HIV co-localized with CD4 and appeared less membrane associated in CD4-SE cells, whereas it remained predominantly on the surface of CD4-SA cells. In CD4-SA cells, intracellular HIV accumulated in EEA1+ and TfR+ compartments (Fig 9), similarly to WT-CD4 cells (Fig 4), whereas HIV accumulated in Lamp-1+ and lysotracker+ compartments in CD4-SE cells (Fig 10), similarly to CD4-DEC cells and CD4-Lamp cells (Fig 5). To test the functional consequences, we transduced HEK-Blue cells with CD4-SA and CD4-SE, as compared to CD4-WT, to measure NF-κB activation, as above. Whereas HIV did not induce NF-κB activation in CD4-WT and CD4-SA expressing cells, it induced NF-κB activation in CD4-SE expressing cells (Fig 11). Thus, CD4 phosphorylation on Ser408 appears to target CD4 and HIV to late endosomes/ lysosomes, whereas it is routed to early endosomes in its absence (CD4-SA and CD4-WT). Poor specificity of available anti-phospho-Ser408 CD4 antibodies precluded the possibility of studying the phosphorylation state of Ser408 CD4 in primary pDCs after HIV activation.

### Trafficking of TLR agonists in pDC is TLR-independent

While our data strongly suggest that HIV trafficking and subsequent immune signaling in pDC is driven by HIV envelope/CD4 interactions, we tested an alternative hypothesis of pDC signaling, that the strength of TLR signaling in the early sorting endosomes determines the trafficking and subsequent signaling of TLR agonists. Previous work showed that TLR signaling in other cell types accelerates endosomal maturation through TLR-induced p38 mitogen-activated protein kinase (p38) signaling, as the absence of a TLR/MyD88 signal diminishes phagosome maturation [44]. TLR activation also activates lysosomal function in myeloid DC, which could further influence TLR signaling pathways [45, 46]. According to this model, strong TLR triggering in the early endosome would accelerate endosomal maturation, and endocytosed viruses or oligonucleotides would rapidly reach late endosomes for NF-κB signaling [25, 33]. Using murine pDC differentiated from TLR7^-/-^ and TLR9^-/-^ bone marrow, we tested this hypothesis. As HIV cannot be used in murine immune systems, we tested TLR9 agonists, focusing on CpGB. CpGB has been shown to rapidly traffic to late endosomes/ lysosomes in mouse and human pDC [25, 26], and it induces strong NF-κB signaling and pDC maturation [22]. According to this model where TLR signaling induces endosomal maturation, CpGB would traffic rapidly to lysosomes in wild type (WT) or TLR7^-/-^ pDC, but would fail to traffic to lysosomes in murine TLR9^-/-^ pDC. Because MyD88 knockout DCs lacking TLR signaling have been described as having an altered constitutive rate of endosomal maturation [44, 47], we also constructed a control oligonucleotide lacking stimulatory CpG motifs (due to inversion of the CpG motif into a GpC dinucleotide) which lacks TLR triggering activity. Wild type, TLR7^-/-^, and TLR9^-/-^ murine pDC were derived from bone marrow with Flt3 ligand, as previously described [48] and purified >85% (S3A Fig). pDCs were incubated with FAM-labeled CpGB or nonactivating FAM-labeled GpC control and were imaged by flow cytometry and live microscopy. We first confirmed the specificity of the various pDC TLR knock out cells by evaluating the expression of the maturation molecule CD86 after overnight incubation of wild type, TLR9^-/-^, and TLR7^-/-^ pDC with TLR7 agonist R848 and TLR9 agonist FAM-CpGB. As expected, R848 matured WT and TLR9^-/-^ pDC but did not mature TLR7^-/-^ pDC, whereas FAM-CpGB matured WT and TLR7^-/-^ pDC but did not mature TLR9^-/-^ pDC. pDC stimulated with FAM-GpC did not mature WT pDC due to the lack of CpG immunostimulatory motifs (S3B Fig). We then monitored trafficking of FAM-CpGB to lysosomes in WT, TLR7^-/-^ and TLR9^-/-^ pDC by microscopy, by measuring co-localization with Lysotracker Red. Across a z stack spanning the midplane of the cell, we observed that both FAM-CpGB in WT, TLR7^-/-^ and TLR9^-/-^ pDC (S4A and S4C Fig), and FAM-GpC in WT pDC (S4B and S4D Fig), rapidly and extensively trafficked to lysosomes within 15-20min. Thus, TLR activation did not affect intracellular trafficking of TLR agonists in murine pDC, suggesting that an alternative model also exists for human pDC. Altogether, these data demonstrate that divergent HIV-1 sensing by pDC is mediated by CD4-HIV envelope interactions.

## Discussion

Although many receptors and signaling pathways have been shown to modulate TLR signaling pathways in pDC [49], it is likely that the functional outcome of TLR signaling is determined upstream and early on. The spatiotemporal model of TLR signaling [25] was proposed to account for the functional flexibility of pDC in response to TLR signaling, and argues that the surface or intracellular localization of TLR signaling initiation determines which downstream signaling pathway is triggered, with differing functional outcomes [50]. This is because each compartment is associated with different adaptors and signaling platforms, specialized in inflammatory cytokine secretion and NF-κB activation, or signaling through IRF7 and IFN production [25, 33, 50]. As HIV steadily traffics to early endosomes in pDC, a compartment associated with IFN signaling [25], our goal was to understand how early HIV trafficking is regulated in pDC and how it affects pDC functional response.

We studied whether HIV trafficking in pDC involves envelope-receptor interaction and targeting signals in endocytic receptors. A hybrid virus was constructed, where HIV envelope was replaced by influenza hemagglutinin envelope, while maintaining all other HIV structural components unchanged (HA-HIV). In contrast to HIV, influenza is rapidly endocytosed by pDC and triggers a strong NF- κB activation, secretion of inflammatory cytokines, and maturation of pDC [22, 51]. Strikingly, HA-HIV was rapidly routed to late endosomes/ lysosomes in pDC, contrary to HIV with its native envelope. Furthermore, it induced early secretion of inflammatory cytokines and strong pDC maturation, in a manner and kinetics similar to influenza virus. This demonstrates that virus envelope directly determines HIV trafficking and pDC phenotype. Despite different structural components and nucleic acids, HA-HIV and influenza induced a similar functional response in pDC, which strengthens the importance of viral envelope in determining pDC phenotype. This latter extended to the unresponsiveness of pDC to further stimulation, whereas HIV stimulated pDC could be re-stimulated to produce IFN. HA-HIV triggers IFN secretion, although at lower levels than Flu itself. This may be due to kinetics differences in trafficking to late endosomes and activation of negative signaling pathways or exhaustion. Trafficking to late endosomes, NF-κB activation and pDC maturation correlates with a state of refractoriness, likely established early during stimulation, and already evidenced by a global cytokine shutdown after a few hours. As shown in Fig 1A, this shutdown occurs earlier for HA-HIV than for Flu, at the time when IFN secretion is starting to be amplified. It is likely HA-HIV traffics significantly faster to late endosomes and triggers early cytokine shutdown.

Another example of HIV pseudotyping is VSV-G pseudotyped HIV, where VSV-G from vesicular stomatitis virus is used to allow HIV uptake and infection of many cellular subtypes. The putative receptor for VSV-G has been recently suggested to be LDL receptor [52], which traffics mainly to recycling endosomes but rarely to lysosomes. In accordance with this trafficking pattern and localization in early endosomes, VSV-G-pseudotyped HIV behaves mostly like HIV with a native envelope to trigger high levels of IFN but little pDC maturation [12]. In addition to demonstrating that viral envelope determines HIV localization and pDC phenotype, HA-pseudotyped HIV may also provide a tool to study HIV antigen presentation and vaccine design [53], as it enhances expression of MHC and co-stimulatory molecules on pDC, and influenza itself triggers a developmental program suited for antigen presentation in pDC [21, 54].

If the nature of the viral envelope dictates HIV trafficking in pDC, it may be due to intracellular targeting motifs present in the viral receptor(s) [38]. HIV is mainly taken up through CD4 in pDC, and we tested whether altering the intracytoplasmic domain of CD4 would affect HIV trafficking and TLR signaling. Indeed, we observed that swapping CD4 intracytoplasmic domain for DEC205 or Lamp1 intracytoplasmic domain dramatically re-routed HIV into late endosomes/ lysosomes in CD4 expressing cells, whereas intracellular HIV was localized predominantly in early endosomes in cells expressing native CD4. DEC205 and Lamp1 contain lysosomal targeting motifs which are likely responsible for constitutive CD4 and HIV targeting to late compartments. In addition, redistribution of HIV to late endosomes was accompanied by activation of NF-κB, not observed when HIV accumulates in early endosomes, again consistent with the spatiotemporal model of TLR signaling. CD4 contains a dileucine motif in its intracytoplasmic domain, which is essential for CD4 endocytosis [36]. In addition, two adjacent Serines, Ser408 and Ser415, can be phosphorylated and impact CD4 endocytosis and distribution. Completely deleting these motifs by removing the whole CD4 intracytoplasmic domain indeed almost completely abrogated CD4-mediated HIV endocytosis. However, phosphorylation of Ser408 not only enhances CD4 endocytosis [36], but also redirects CD4 to lysosomal compartments [43]. Ser408 phosphorylation enhances its association with clathrin Adaptor protein AP-1 and AP-2 [36]. In our experiments, mutating Ser408 to Glutamic acid (CD4-SE) to mimic Ser408 phosphorylation, induced a complete redistribution of CD4 and HIV into lysosomes (Fig 10) suggesting that in pDC, HIV traffics by default to recycling endosomes due to the CD4 dileucine motif, in the absence of Ser408 phosphorylation. These results are supported by our CD4 internalization studies where we found that HIV-activation of pDC does not seem to alter CD4 internalization, as compared to PMA-activation. Similarly, in HIV infected cells, Nef triggers endocytosis and degradation of CD4 through a dileucine based motif [55, 56] and CD4 endocytosis and targeting to lysosomes are encoded in different regions of the Nef protein [56, 57].

The relatively stable localization of HIV in early endosomes, observed for as long as 18 to 24h, remains unexplained. Furthermore, we observed strong co-localization of CD4 with HIV throughout the course of the study. Although the interaction between CD4 and HIV envelope may be stable enough to maintain CD4-HIV co-localization for a prolonged period of time, other explanations are possible. A recent study described in detail how endocytosis of HIV is coupled to dynamin-dependent endocytosis and partial fusion with plasma and endosomal membrane [58], which may tether HIV envelope and CD4 to the endosomal membrane in the absence of fusion. Furthermore, pDC possess specialized large perinuclear intracellular stores of MHC-I molecules, with characteristics of recycling endosomes in immature pDC, that can be used as sites for rapid MHC-I loading and peptide presentation [21]. These intracellular stores may represent the stable compartment in which HIV accumulates in pDC, and prolonged localization in these early recycling endosomes may ultimately have important consequences for HIV antigen presentation. pDC may harbor HIV in these structures until activated by a maturation stimulus. Indeed, pDC are capable of HIV antigen cross-presentation [17], and cross-presentation is strongly enhanced by maturation-inducing stimuli [59, 60]. Upon influenza activation, stored MHC-I molecules are translocated to the cell surface for efficient cross-presentation by pDC [21], indicating that the process of maturation drives antigen presentation.

On the other hand, the non-acidic environment and limited access to MHC-II compartments may prevent efficient MHC-II peptide generation and association with MHC-II molecules. In addition, MHC-II clustering and antigen presentation by pDC is dependent on NF-κB [51, 61], which HIV weakly induces due to localization in early endosomes. The lack of pDC maturation induced by HIV might prevent effective cross-presentation and MHC-II restricted presentation, due to localization in early endosomes and weak NF-κB activation. Thus, the particular compartmentalization of HIV can affect HIV antigen presentation. As shown here, HA-pseudotyped HIV, which traffics to late endosomes and activates NF- κB, may serve as a tool to enhance cross-presentation of HIV antigens by pDC.

We also tested whether TLR signaling itself alters maturation of endocytic compartments in pDC as was previously demonstrated in the case of murine macrophages, where TLR/ MyD88 signaling induced marked phagosome maturation, possibly through p38 MAP kinase activation [44]. We tested this model in murine pDC, and compared trafficking of CpGB in WT, TLR7^-/-^ or TLR9^-/-^ pDC. We did not observe any difference in trafficking of CpGB in these conditions, and internalized CpGB was rapidly found within late endosomes/ lysosomes whether TLR signaling had occurred or not. As MyD88^-/-^ cells have altered endosomal maturation [47], we also used a non-stimulatory type B oligonucleotide (lacking immunostimulatory CpG motifs) in WT pDC. However, its trafficking was identical to the stimulatory CpGB oligonucleotide. Furthermore, as described previously [44, 62], the lack of TLR signaling, as with nonstimulatory GpC, decreased the speed of CpG endocytosis (S4E Fig), however all oligonucleotides localized identically. These data argue against the above model, whereby rate of endocytosis and/ or agonist potency drive its intracellular localization, and establish trafficking as independent of TLR signaling in pDC [47, 63].

Using purified human pDCs and viruses, we demonstrate that HIV trafficking in pDC at early timepoints is determined by the initial envelope-receptor (CD4) interaction, and is regulated by receptor targeting motifs. Whether this is still valid in the case of cell-associated virus, this remains to be determined. Engineering of viral envelope for increased pDC maturation and antigen presentation (e.g. HA-HIV) or for increased IFN secretion [64] may prove useful for vaccine design and modulation of chronic immune activation in HIV disease.

## Materials and Methods

### Human pDC purification

PBMCs were separated on Ficoll-Hypaque (Amersham Biosciences) from buffy coats (New York Blood Center). pDC were purified by BDCA-4 magnetic bead separation (Miltenyi Biotec) as described previously [12], with a purity ranging from 80 to 95%. Cells were cultured in RPMI 1640 Glutamax (Invitrogen) with 5% PHS (Innovative Research, MI), gentamycin, and HEPES.

### HIV, pseudotyped HIV virions, and influenza virions

HIV-1MN (X4-tropic) were produced at the AIDS Vaccine Program, National Cancer Institute as previously described [12, 23, 65]. Plasmids encoding JRFL HIV-1 envelope, JOTO HIV-1 envelope, REJO HIV-1 envelope, pNL43-ΔEnv-vpr+-luc+ and pCAGGS (human airway trypsin-like protease to cleave HA0 to HA1 and HA2) were provided by Carol Weiss (FDA, Silver Spring, MD), plasmids encoding CMV/R Influenza H1(A/PR8/8/34)(VRC 7702), CMV/R influenza A/PR/8/1934 NA (VRC 9776), CMV/8R A/Thailand/1Kan-1/2004 H5 (VRC 7705), and CMV/8R A/Thailand/1Kan-1/2004 NA (VRC 7708), and were provided by Gary Nabel and Chih-Jen Wei(Vaccine Research Center, NIH, Bethesda, MD). Plasmids encoding vpr-gfp and MN HIV pNL4-3 Δvpr were obtained from David Ott and Jeffrey Lifson (AIDS Vaccine Program, Frederick, MD). Plasmids encoding HIV Gag-iGFP JRFL were obtained from Benjamin Chen (Mount Sinai School of Medicine, NY, NY). The influenza hemagglutinin viral pseudotypes were generated by calcium phosphate co-transfection of 3.0x10^6^ HEK cells in a 10cm^2^ dish with 10ug HIV core (pNL43-ΔEnv-vpr+-luc+), 400ng hemagglutinin envelope (VRC 7702, VRC 9776, VRC 7705, or VRC 7708), 100ng PR8 NA (VRC9776), and 100ng HAT pCAGGS, with media change after 6 hours, and viral harvest at 48 hours. HIV pseudotypes were generated by calcium phosphate co-transfection of 3.0x10^6^ HEK cells in a 10cm^2^ dish with 10ug HIV core (pNL43-ΔEnv-vpr+-luc+) and 6ug Env in pCDNA3.1 (e.g. REJO, JOTO, JRFL). HIV Gag-iGFP is a full-length molecular clone of HIV derived from pNL4-3 that packages GFP inserted into the Gag protein between the MA and CA domains of Gag, with JRFL Env cloned into the place of the NL4-3 Env. To generate Gag-iGFP virions for CD4-expressing HEK experiments, 20ug of plasmid was transfected using the calcium phosphate method, with media change at 6 hours and transfection for 48 hours. For all viruses, transfection supernatants were filtered through a 0.45uM membrane, pelleted through a 20% sucrose cushion at 25,000g for 2hrs at 4°C. Pelleted viruses were resuspended in PBS, aliquoted, and stored at -80°C until use. HIV virions were quantified using p24 ELISA (AIDS Vaccine Program) and HA-HIV viruses were quantified using turkey hemagglutination inhibition assay for hemagglutination unit (HAU) as well as p24 ELISA. PR8 influenza was provided by David Levy (NYU) and influenza packaging GFP was provided by Jesse Bloom (Fred Hutchinson Cancer Research Center, Seattle, WA) as previously described [34, 35] and were quantified by using turkey hemagglutination inhibition assay for hemagglutination unit.

### Human pDC activating agonists

Purified pDC were stimulated at 50,000 cells/100uL media at 37° C with 5% CO_2_ with: MN HIV 300ng (AIDS Vaccine Program, National Cancer Institute), HIV pseudotyped HIV envelopes 300ng, type B CPG oligodeoxyribonucleotide (CpGB) 2 ug 5’ T*C*G*T*C*G*T*T*T*T*G*T*C*G*T*T*T*T*G *T*C*G*T*T*-3’ where asterisks indicate a phosphorothioate bond (IDT), Resiquimod (R848) 10μM (3M Corporation, St. Paul, MN), influenza virus PR8 HAU 10 heat inactivated at 56°C for 30 minutes in a water bath, or HA-HIV HAU 10. For intracellular staining, brefeldin A was added after 30 minutes, 2 hours, 6 hours, or 12 hours; at 24 hours cells were washed, fixed, and stained with PE-conjugated CD123 PE (BD Biosciences), APC-conjugated TNFα (eBioscience), and FITC-conjugated IFN-α (BD Biosciences) in 0.05% saponin, and analyzed by FACS. Following incubation of pDCs (50,000 cells/100uL) with HIV MN and HIV JRFL, influenza, and HA-HIV, culture supernatants were tested for IFN and TNF by IFNα by ELISA (PBL Interferon Source) and human inflammatory kit CBA(Abcam), respectively, following manufacturer instructions. For surface staining after overnight incubation, cells were stained with CD123 PE, CD86 APC, and HLA-DR PerCP(BD Pharmigen), washed, fixed with 4%PFA, and analyzed by FACS. For IFNα restimulation experiment, pDCs were incubated overnight with influenza, HIV, HA-HIV, or CpGB, washed, and then incubated again with the same agonists. Culture supernatants were tested after the first and second overnight incubation for IFNα by ELISA (PBL Interferon Source).

### CD4 mutants construction and cell lines

CD4 expression plasmid (pcDNAI) was provided by Nathaniel Landau (NYU, NY, NY). It was used as a template to mutate CD4 by overlapping PCR. Mutated CD4 were then inserted into pLenti vectors, and lentiviruses were produced using Mirus TransIT co-transfection of 3.0x10^6^ HEK cells in a 10cm^2^ dish for 72 hours with 1.45 ug VSVg, 2.05 ug RSR-Rev, 2.9 ug pMDL plasmids (provided by Dr Landau) and 8.7 ug pLenti-CD4 constructs (WT, STOP, DEC, LAMP, SE, SA). CD4-STOP was encoded of the first 425 amino-acids, including the transmembrane domain of CD4. CD4-DEC contained the full extracellular domain of CD4 (397 amino acid in the immature form) and the transmembrane and intracytoplasmic domain of DEC205 (56 amino acids). CD4-Lamp encoded the full extracellular and transmembrane domain of CD4, and 12 amino acids of Lamp intracytoplasmic domain. CD4-SE and CD4-SA was identical to CD4 WT, except for a mutation of Serine 408 (mature form) for glutamate and alanine, respectively. CD4-expressing HEK cell lines were generated by infecting 3.0x10^6^ HEK cells in a 10cm^2^ dish with lentivirus transfection supernatants and CD4 expression was maintained in the presence of puromycin.

### Murine pDC isolation

C57BL/6 wild type, TLR7^-/-^ and TLR9^-/-^ double-knockout mouse femurs were provided by B. Pulendran, Emory University. Mice were maintained in specific-pathogen-free conditions at the Emory Vaccine Center vivarium in accordance with all animal protocols reviewed and approved by the Institute Animal Care and Use Committee of Emory University. Bone marrow cells were isolated by flushing femurs with PBS supplemented with 2% heat inactivated FBS. BM cells were resuspended in Tris-ammonium chloride at room temperature for 1 minute to lyse RBC, washed, then cultured in RPMI 1640 Glutamax (Invitrogen) with 10%FBS, 1nM sodium pyruvate, 10mM HEPES buffer, 100 units/mL penicillin, 100ug/mL streptomycin, 2mM L-glutamine, 1% MEM nonessential amino acids. BM cells were cultured for 8 days at 1 × 10^6^ cells/ml in 24-well plates in culture medium supplemented with 200 ng/ml recombinant murine Flt-3 ligand (Peprotech) as previously described [48]. After 8 days pDCs were purified from BM cells using the mouse plasmacytoid dendritic cell isolation kit II (Miltenyi) with a purity ranging from 85 to 95% and cells were phenotyped by CD11c PerCP and PDCA1 APC (BD Pharmigen).

### Murine pDC stimulation

Purified murine pDC (WT, TLR7^-/-^, and TLR9^-/-^) were stimulated overnight at 50,000 cells/100uL media at 37°C with 5% CO_2_ with R848 10μM (3M), 5’FAM-CpGB 5’ TCGTCGTTTTGTCGTTTTGTCGTT-3’ 2 ug (IDT), or 5’FAM-GpC 5’-TGCTGCTTTTGTGCTTTTGTGCTT-3' 2ug (IDT), both with phosphodiester backbones, and were stained with CD11c PerCP, PDCA1 APC, and CD86 PE (BD Pharmigen).

### Live microscopy of murine pDC and human pDC

Purified murine pDC (200,000 cells/200uL) were stimulated in 0.01% Poly L-lysine (Sigma) coated 8 chamber polystyrene vessel tissue culture treated glass slides (CultureSlides BD Falcon) in 10% FBS culture media (200uL) with FAM-CpGB 2ug or FAM-GpC 2ug and stained with 1uM lysotracker (Invitrogen). Cells were imaged by live microscopy for one hour using the Advanced Precision PersonalDV Imaging system, with temperature (37°C) and C0_2_ (5%) humidity control, at a size of 512 X 512 pixels and a bit depth of 16 using a 60X, 1.4 N.A. oil objective lens. Images were deconvoluted using the DeltaVision deconvolution system and analyzed using ImageJ. Purified human pDC (50,000 cells/50uL) were stimulated in Fibronectin (Corning) coated Ibidi imaging chambers μ-Slide VI^0.4^ (Ibidi, Madison, WI) in 5% PHS culture media (200uL) with GFP PR8 influenza 10 HAU, GFP HA-HIV (10HAU/500ng p24) or GFP JRFL pseudotyped HIV 500ng p24 and stained with 1uM lysotracker (Invitrogen). Live imaging was carried out after 30 minutes, 2–4 hours, or after overnight stimulation of cells, using a Yokogawa CSU-X1 spinning disk mounted on a Zeiss AxioObserver Z1 and controlled by MetaMorph under conditions that were reproduced across all experiments. 488nm and 561nm laser lines were generated by a Prairie Technologies Aurora solid state laser and fluorescence and brightfield (phase) images were captured with a Hamamatsu EM-CCD C9100 digital camera set at an EM gain of 11 MHz, a size of 512 X 512 pixels and a bit depth of 16 using a 63X, 1.4 N.A. oil objective lens. Temperature (37°C), C0_2_ (5%), and humidity were controlled using a Tokai Hit incubator.

### CD4 blocking

CD4 expressing HEK cells were incubated (200,000 cells/200 μl) with purified NA/LE mouse anti-human CD4 (10 μg/ml; BD Pharmigen) or isotype control purified NA/LE mouse IgG_1_κ for 30 minutes. HIV Gag-iGFP was then added, and cells were placed back in culture for 18 hours. Cells were washed, fixed with 4% PFA, and analyzed by FACS.

### Confocal microscopy of CD4-expressing HEK cells

Purified human pDC (100,000) were incubated for 18 hours with GFP PR8 influenza 10 HAU, GFP HA-HIV (10HAU/500ng p24) or GFP JRFL pseudotyped HIV (HIV Gag-iGFP JRFL) 500ng p24 in 8 chamber polystyrene vessel tissue culture treated glass slides (CultureSlides BD Falcon) in 10% FBS (Gibco) culture media (200uL) then cells were washed x2, fixed with 4% paraformaldehyde, permeabilized with 0.1% Triton X-100, blocked with 0.5% BSA in PBS, stained with mouse anti-EEA1 (0.5 ug; BD biosciences) at 4°C overnight, washed x 2 then stained with donkey anti-mouse TRITC (Jackson Immunoresearch) for one hour at RT, then washed, dried, and mounted in DAPI Anti-fade GOLD (Vector labs). Alternatively pDCs were incubated with media, phorbol 12-myristate 13-acetate 100ng/mL (PMA), HIV, or PMA and HIV for 4 hours then cells were washed x2, fixed with 4% paraformaldehyde, permeabilized with 0.1% Triton X-100, blocked with 0.5% BSA in PBS, and then stained with CD4 PE (BD Pharmigen) for one hour, washed x2, dried, and mounted in DAPI Anti-fade GOLD (Vector labs). HEK cells transduced with CD4 mutants (25,000) were incubated overnight in 8 chamber polystyrene vessel tissue culture treated glass slides (CultureSlides BD Falcon) in 10% FBS (Gibco) culture media (200uL). iGFP HIV 500ng was added to culture media for 2–4 hours then cells were washed x2, fixed with 4% paraformaldehyde, permeabilized with 0.1% Triton X-100, blocked with 0.5% BSA in PBS, and then stained with CD4 PE (BD Pharmigen) for one hour. Alternatively, cells were stained with mouse anti-EEA1 (0.5 ug; BD biosciences), mouse anti-transferrin receptor (0.5ug; Invitrogen), mouse anti LAMP-1 H4A3 (0.5ug; Developmental Studies Hybridoma Bank University of Iowa) at 4°C overnight, washed x 2 then stained with donkey anti-mouse TRITC (Jackson Immunoresearch) for one hour at RT, then washed, dried, and mounted in DAPI Anti-fade GOLD (Vector labs). Fixed cells were imaged using a Zeiss LSM 880 confocal microscope configured to generate laser lines at 488nm and 561nm, as well as transmitted light. Images were scanned using a 100X, 1.46 N. A. oil objective lens at a size of 1024 X 1024 pixels and a bit depth of 12. Images were captured using sequential (multitrack) acquisition to avoid bleedthrough of signal between channels.

### Colocalization measurements

Colocalization of fluorescently labeled ligands (green) with endosomal markers or CD4 receptor (red) were analyzed quantitatively using JACoP plugin on ImageJ software or MetaMorph colocalization analysis. Image pair channel files of single image mid-planes were opened in as separate 16-bit (grey scale) image files. Individual cell regions were identified using the corresponding brightfield image, images were thresholded, and colocalization analysis was performed using the MetaMorph “measure colocalization” application, which measures the localization correlation between corresponding pixels in two paired images and provides the Manders correlation [66]. 50–100 cells were analyzed to generate the individual coefficients and data was plotted using GraphPad Prism software.

### HEK-Blue

CD4-expressing HEK-Blue hTLR cell lines were generated by infecting 3.0x10^6^ HEK-Blue hTLR cells in a 10cm^2^ dish with CD4 lentivirus transfection supernatants. Stable CD4 expression of cell lines (WT, STOP, DEC, LAMP, SA, and SE) were cultured in the presence of puromycin and CD4 expression was verified by FACS and microscopy. 200,000 cells were placed in HEK-blue culture media overnight (200uL) with CpGB, HIV, or R848 and NF-KB activation was measured using the HEK-blue detection system, with blue color depicting NF-κB activation, as measured by ELISA according to manufacturer specification (Invivogen).

### Statistics

Statistical significance was determined by the unpaired Student’s t test and analysis of variance.

## Supporting Information

S1 Fig HIV pseudotyped with influenza hemagglutinin envelope activates signaling pathways in pDC similarly to influenza. (A) Human purified pDC incubated with virions overnight and stained for maturation marker CD86. Data are from 1 experiment, representative of 3 independent experiments. pDC were incubated with media (-), HIV (HIV backbone pNL43-ΔEnv-vpr+-luc+ pseudotyped with X5 HIV envelopes (JRFL, REJO, JOTO) as compared to HIV backbone pNL43-ΔEnv-vpr+-luc+ pseudotyped with hemagglutinin envelopes H1 and H5, as compared to R848 and Flu. (B) Interferon-alpha (IFN) production after overnight incubation of pDC with media (-), MN HIV, or HIV backbone pNL43-ΔEnv-vpr+-luc+ pseudotyped with X5 HIV envelopes (JRFL, REJO, JOTO). IFN was measured in the culture supernatants by ELISA. Bar graphs represent 3 experiments with mean ± SD.
(TIF)

S2 Fig Viral-envelope directs intracellular trafficking of HIV virions in pDC. (A, B) Representative images from live microscopy showing brightfield images (BF) and staining for lysotracker (Lyso) of cells incubated with GFP-influenza (Flu), GFP-HA-HIV (HA-HIV), or GFP-HIV (HIV) for a single confocal z stack with scale bar = 20μm and inset (3X) at (A) 2–4 hours and (B) after 18 hours. Overlay of paired images and inset (3X) shown. Data representative of 3 experiments. Magnification X63. Graphs depict % colocalization of 50 cells shown for lysotracker/lysosome (Manders’ coefficient) with virions at (C) 2–4 hours with mean ±SD comparing Flu 90.48% ±13.83% to HA-HIV 91.64% ± 16.50% to HIV 0.00% ± 0.00% and (D) after 18 hours with mean ±SD comparing Flu 92% ±16.48% to HA-HIV 86.24% ± 18.39% to HIV 15.54% ± 31.20%, unpaired Student’s t test comparing Flu to HIV and HA-HIV to HIV, *p<0.001.
(TIF)

S3 Fig pDC generated from Flt3 ligand-supplemented BM cultures from WT, TLR7^-/-^, and TLR9^-/-^ mice. (A) Representative scatter plot of purification schema (B) Purified murine pDC were incubated overnight with R848, CpGB, or GpC. FACS demonstrating maturation as assessed by CD86 expression, Unstimulated cells (open histogram), stimulated cells (filled histogram). Data representative of 3 experiments.
(TIF)

S4 Fig Trafficking of TLR agonists in pDC is TLR-independent. Functional responses of murine pDC generated from Flt3 ligand-supplemented BM cultures from WT, TLR7^-/-^, and TLR9^-/-^ mice. (A-E) Murine BM purified pDC 2–4 hours post incubation with FAM-CpGB or FAM-GpC. (A) Images from live microscopy showing representative staining for lysotracker (Lyso) of cells incubated with FAM-CpGB for a single confocal z stack. (C) Graphs depict % colocalization shown for lysotracker (Manders’ coefficient) for 100 cells with mean ± SD comparing WT with TLR7-/- and WT with TLR9-/- (98.09±0.80 vs 96.62±0.99) and (98.09±0.80 vs 96.45±0.98). (B) Images from live microscopy showing representative staining for lysotracker (Lyso) of cells incubated with FAM-GpC for a single confocal z stack. (D) Graphs depict % colocalization shown for lysotracker (Manders’ coefficient) for 100 cells with mean ± SD comparing CpGB with GpC (91.90±1.73 vs 93.28±1.51). Data representative of 3 experiments. Results represented with mean bar; N.S., not statistically significant. Magnification X60. (E) Purified murine pDC (WT) were incubated overnight with CpGB-FAM or GpC-FAM. FACS demonstrating uptake as assessed by FAM fluorescence, Unstimulated cells (open histogram), stimulated cells (filled histograms). Data representative of 3 experiments.
(TIF)
