# Supplementary figures and images for: CD4 Receptor is a Key Determinant of Divergent HIV-1 Sensing by Plasmacytoid Dendritic Cells
Source: PLoS Pathog. 2016 Apr 15;12(4):e1005553. doi: 10.1371/journal.ppat.1005553 (PMC4833349; doi:10.1371/journal.ppat.1005553)

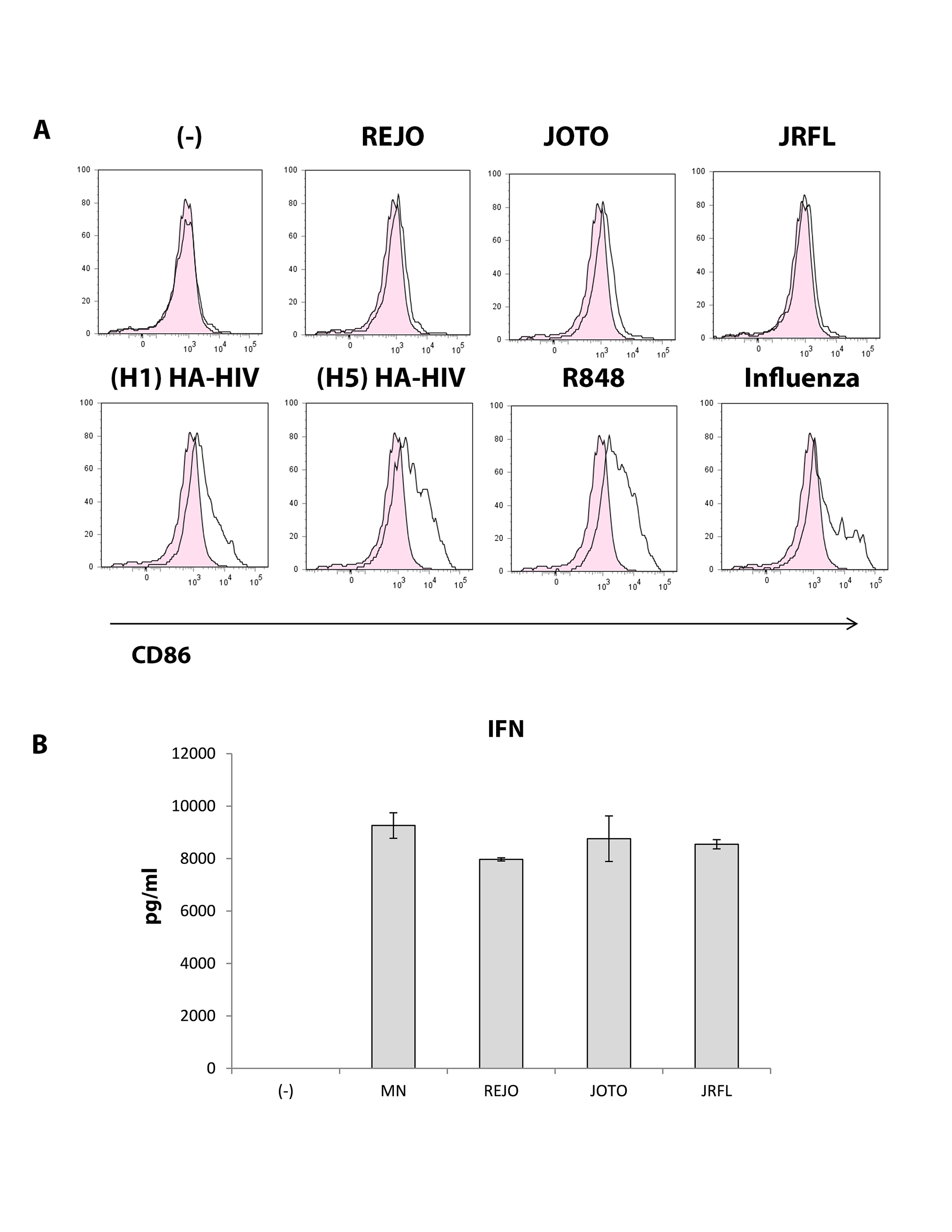

Supplement: S1 Fig — (A) Human purified pDC incubated with virions overnight and stained for maturation marker CD86. Data are from 1 experiment, representative of 3 independent experiments. pDC were incubated with media (-), HIV (HIV backbone pNL43-ΔEnv-vpr+-luc+ pseudotyped with X5 HIV envelopes (JRFL, REJO, JOTO) as compared to HIV backbone pNL43-ΔEnv-vpr+-luc+ pseudotyped with hemagglutinin envelopes H1 and H5, as compared to R848 and Flu. (B) Interferon-alpha (IFN) production after overnight incubation of pDC with media (-), MN HIV, or HIV backbone pNL43-ΔEnv-vpr+-luc+ pseudotyped with X5 HIV envelopes (JRFL, REJO, JOTO). IFN was measured in the culture supernatants by ELISA. Bar graphs represent 3 experiments with mean ± SD. (TIF) [file ppat.1005553.s001.tif]

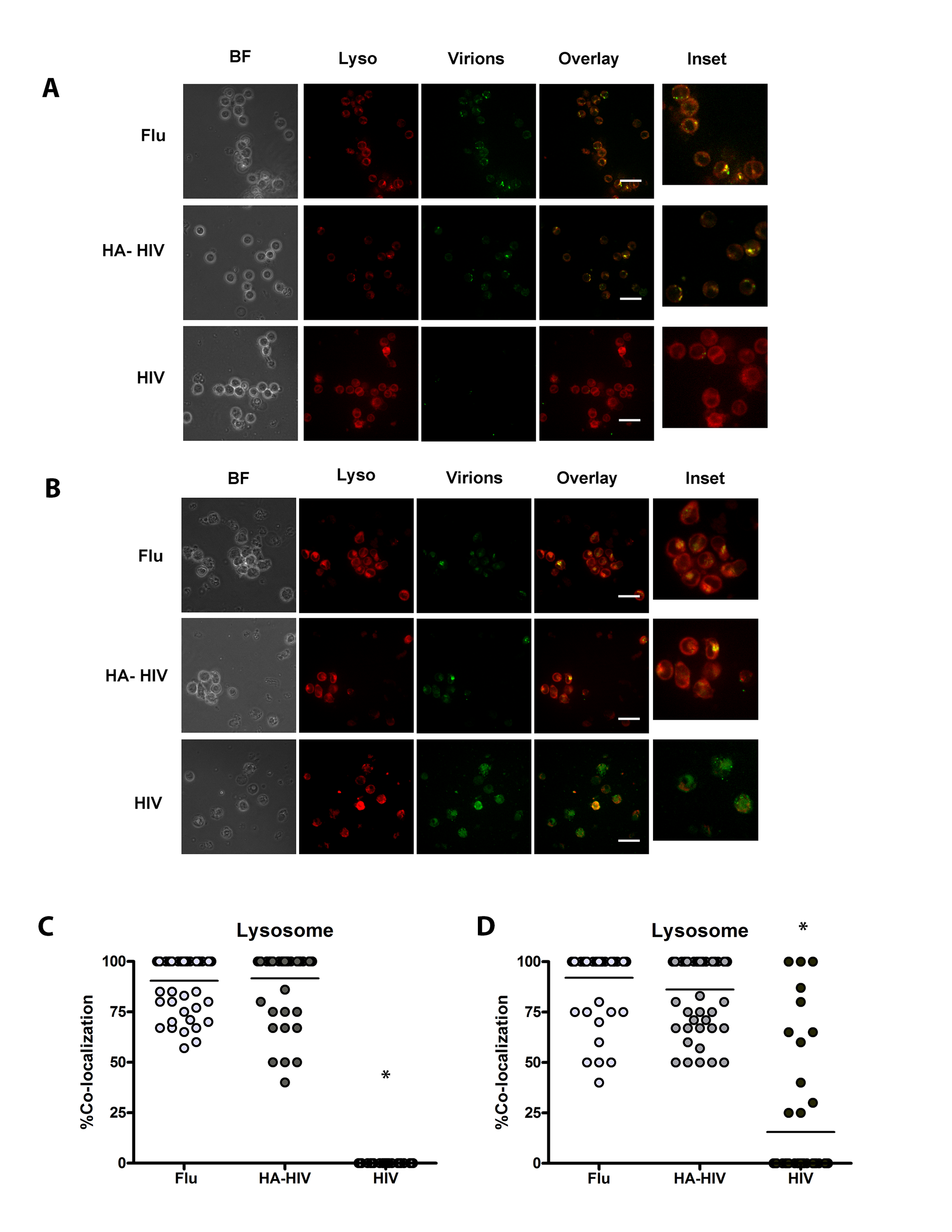

Supplement: S2 Fig — (A, B) Representative images from live microscopy showing brightfield images (BF) and staining for lysotracker (Lyso) of cells incubated with GFP-influenza (Flu), GFP-HA-HIV (HA-HIV), or GFP-HIV (HIV) for a single confocal z stack with scale bar = 20μm and inset (3X) at (A) 2–4 hours and (B) after 18 hours. Overlay of paired images and inset (3X) shown. Data representative of 3 experiments. Magnification X63. Graphs depict % colocalization of 50 cells shown for lysotracker/lysosome (Manders’ coefficient) with virions at (C) 2–4 hours with mean ±SD comparing Flu 90.48% ±13.83% to HA-HIV 91.64% ± 16.50% to HIV 0.00% ± 0.00% and (D) after 18 hours with mean ±SD comparing Flu 92% ±16.48% to HA-HIV 86.24% ± 18.39% to HIV 15.54% ± 31.20%, unpaired Student’s t test comparing Flu to HIV and HA-HIV to HIV, *p<0.001. (TIF) [file ppat.1005553.s002.tif]

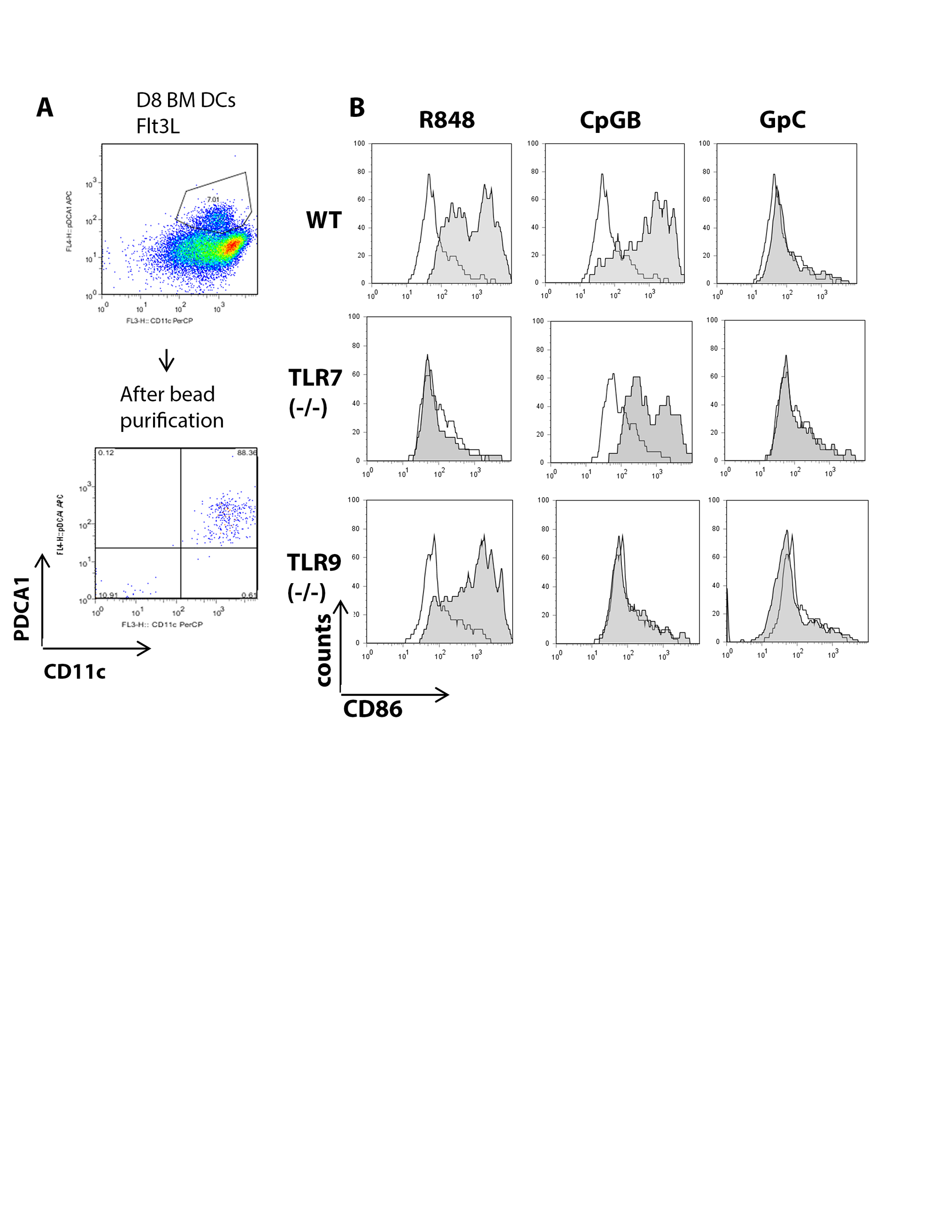

Supplement: S3 Fig — (A) Representative scatter plot of purification schema (B) Purified murine pDC were incubated overnight with R848, CpGB, or GpC. FACS demonstrating maturation as assessed by CD86 expression, Unstimulated cells (open histogram), stimulated cells (filled histogram). Data representative of 3 experiments. (TIF) [file ppat.1005553.s003.tif]

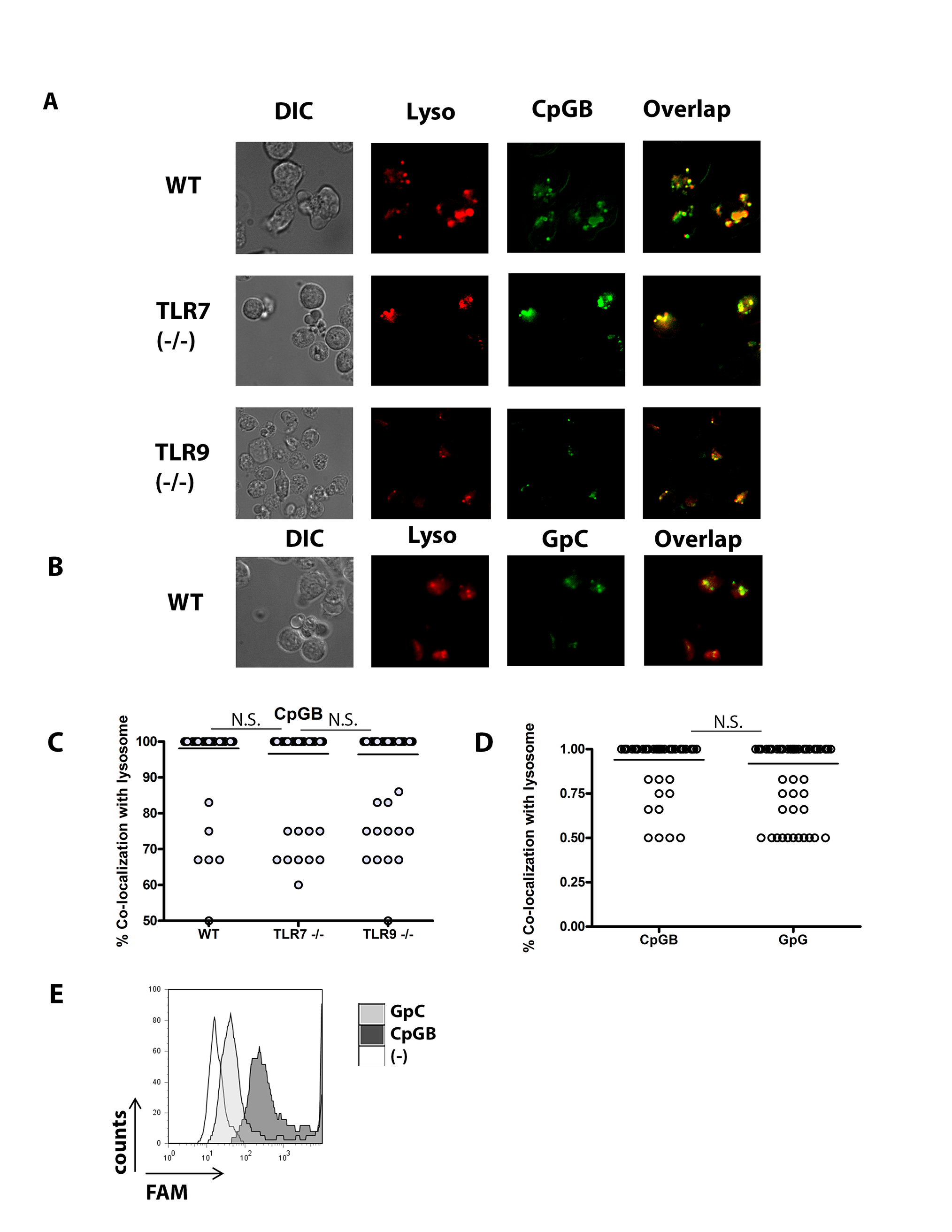

Supplement: S4 Fig — Functional responses of murine pDC generated from Flt3 ligand-supplemented BM cultures from WT, TLR7-/-, and TLR9-/- mice. (A-E) Murine BM purified pDC 2–4 hours post incubation with FAM-CpGB or FAM-GpC. (A) Images from live microscopy showing representative staining for lysotracker (Lyso) of cells incubated with FAM-CpGB for a single confocal z stack. (C) Graphs depict % colocalization shown for lysotracker (Manders’ coefficient) for 100 cells with mean ± SD comparing WT with TLR7-/- and WT with TLR9-/- (98.09±0.80 vs 96.62±0.99) and (98.09±0.80 vs 96.45±0.98). (B) Images from live microscopy showing representative staining for lysotracker (Lyso) of cells incubated with FAM-GpC for a single confocal z stack. (D) Graphs depict % colocalization shown for lysotracker (Manders’ coefficient) for 100 cells with mean ± SD comparing CpGB with GpC (91.90±1.73 vs 93.28±1.51). Data representative of 3 experiments. Results represented with mean bar; N.S., not statistically significant. Magnification X60. (E) Purified murine pDC (WT) were incubated overnight with CpGB-FAM or GpC-FAM. FACS demonstrating uptake as assessed by FAM fluorescence, Unstimulated cells (open histogram), stimulated cells (filled histograms). Data representative of 3 experiments. (TIF) [file ppat.1005553.s004.tif]
